# Chromosomal Rearrangements between Serotype A and D Strains in *Cryptococcus neoformans*

**DOI:** 10.1371/journal.pone.0005524

**Published:** 2009-05-13

**Authors:** Sheng Sun, Jianping Xu

**Affiliations:** Department of Biology, McMaster University, Hamilton, Ontario, Canada; University of Texas-Houston Medical School, United States of America

## Abstract

*Cryptococcus neoformans* is a major human pathogenic fungus that can cause meningoencephalitis in immunocompromised hosts. It contains two divergent varieties, var. *grubii* (serotype A) and var. *neoformans* (serotype D), as well as hybrids (serotype AD) between these two varieties. In this study, we investigated the extent of chromosomal rearrangements between the two varieties, estimated the effects of chromosomal rearrangements on recombination frequencies, and surveyed the potential polymorphisms of the rearrangements among natural strains of the three serotypes. Through the analyses of two sequenced genomes from strains H99 (representing var. *grubii*) and JEC21 (representing var. *neoformans*), we revealed a total of 32 unambiguous chromosome rearrangements, including five translocations, nine simple inversions, and 18 complex rearrangements. Our analyses identified that overall, rearranged regions had recombination frequencies about half of those around syntenic regions. Using a direct PCR screening strategy, we examined the potential polymorphisms of 11 rearrangements among 64 natural *C. neoformans* strains from five countries. We found no polymorphism within var. *neoformans* and very limited polymorphism within var. *grubii*. However, strains of serotype AD showed significant polymorphism, consistent with their hybrid origins coupled with differential loss of heterozygosity. We discuss the implications of these results on the genome structure, ecology, and evolution of *C. neoformans*.

## Introduction

When populations become isolated, genetic differences may accumulate as a result of differential fixation of spontaneous mutations by genetic drift and/or natural selection. Mutations may be classified into two broad types. The first type is small-scale point mutations, including nucleotide substitutions and short insertions or deletions. The second type is large-scale changes that include large duplications, deletions, and chromosomal rearrangements such as inversions and translocations. When large-scale changes occur, the genomic size and gene content of the diverging genomes may remain similar, but the physical locations and/or orientations of certain chromosomal segments can differ among the diverging lineages.

Large-scale genetic changes can arise spontaneously and have been observed in natural populations of many organisms. For example, chromosomal rearrangements are commonly found in bacteria (e.g. see review by HUGHES [1]) as well as in diverse groups of eukaryotic organisms such as the Baker's yeast (*Saccharomyces cerevisiae*), the fruit fly (*Drosophila melanogaster*), the common mosquito (*Anopheles gambiae*), and humans [2]–[4; for recent reviews, see 5–7]. Some of these rearrangements significantly impacted the phenotypes of these organisms. For example, in species of *Drosophila*, inversions have been linked to the variation in a diversity of traits such as body size, tolerance and resistance to extreme temperatures, wing size, female fecundity, and male mating success [6]. In humans, chromosomal rearrangements have been linked to a large number of diseases, including cancer. As a result, chromosomal rearrangements can serve as direct targets for natural selection and be accumulated in different lineages as a result of such selection (or by genetic drift in small populations), contributing to the divergence between lineages. It has been known for a long time that chromosomal rearrangements can also play a critical role in speciation. Specifically, homologous but rearranged chromosomes may not be able to undergo proper pairing and/or disjunction during meiosis, thus can suppress recombination and accelerate divergence and speciation by reducing hybrid fitness [5].

Speciation commonly refers to the complete fixation of alternative alleles in loci involved in reproductive isolation between lineages. These loci, also called isolation loci, can act on a variety of phenotypic and physiological traits to ensure pre-zygotic and/or post-zygotic isolation. However, unless the diverging lineages are allopatric (i.e. they are separated by geographic barriers), the fixation of different alleles at the isolation loci among lineages could be interrupted or eroded by gene flow between lineages through inter-lineage hybridization. During inter-lineage hybridizations, meiosis and recombination could cause introgression of fixed alleles among the lineages, leading to homogenization and preventing lineage divergence. The locations of these isolation loci could influence the rate of fixation. For example, if the isolation loci are located within or tightly linked to rearranged chromosomal regions, their fixation in different lineages can be significantly facilitated. This is because chromosomal rearrangements can suppress recombination during inter-lineage hybridization by interrupting proper pairing between homologous chromosomes during meiosis or by the generation of non-viable progeny because of irregularities in chromosome number (e.g. aneuploidy) and/or chromosome structure (e.g., chromosomes with no or two centromeres, [5]).

Recent studies have shown that both small-scale point mutations and large-scale genome changes can play important roles in shaping the evolutionary history and population structure of closely related lineages/species [8]–[10; for recent reviews, see 5, 11]. Therefore, identifying chromosome rearrangements between diverging lineages and studying the distributions of these rearrangements in natural populations can provide significant information about the evolutionary histories of different lineages and help us understand the population structure and dynamics of the organisms.

*Cryptococcus neoformans* is an opportunistic human fungal pathogen that can cause meningitis, mainly in immuno-compromised patients (e.g. AIDS and transplant surgery patients). In AIDS patients, it has been estimated that *C. neoformans* accounts for up to 15% of total fatality [12]. Because of its medical importance and ease of genetic manipulation, *C. neoformans* has been one of the most extensively studied human fungal pathogens in the past couple of decades. *C. neoformans* contains strains belonging to three serotypes, A, D, and AD. Strains of serotypes A and D are generally haploid and correspond to two varieties, var. *grubii* and var. *neoformans*, respectively; while strains of serotype AD are recent natural hybrids between strains of serotypes A and D and are mostly diploid or aneuploid [13]–[15]. Gene genealogies have suggested that these two varieties have diverged from each other for at least 18.5 million years [16], and DNA sequence divergence between serotype A and D strains is between 10–15% [17]. Despite their significant divergence, mating between strains of the two varieties can occur. However, the viability of meiotic progeny from the hybrid cross is typically low [13]. Furthermore, recombination frequencies across many chromosomal regions were significantly lower in an inter-variety cross than in intra-variety crosses [18]–[20]. These results suggest that certain types of genetic differences between the two varieties compromised meiosis and recombination during inter-variety hybridization and that partial reproductive isolation has been established between the two varieties. At present, the type, number, and distributions of specific genes involved in the partial reproductive isolation between the two varieties remain unknown.

While most population and epidemiological studies of *C. neoformans* have focused on point mutations in DNA sequences, several studies using pulse-field gel electrophoresis (PFGE) have shown that extensive karyotype variation (i.e., chromosome number and size) exists among natural *C. neoformans* strains [21]–[24]. FRASER et al. [25] showed that during the process of constructing the isogenic *C. neoformans* JEC20/JEC21 laboratory strains, telomere-telomere fusion and chromosomal breakage likely had occurred, resulting in a large translocation and segmental duplication in the JEC20/JEC21 genome compared to their parental strains. These studies suggest that chromosomal rearrangements might occur frequently in *C. neoformans*. However, while PFGE can only detect large insertions/deletions and translocations that involve more than one chromosome, it cannot detect intra-chromosomal translocations or inversions.

Since the genomes of two representative strains of *C. neoformans* are available, one for a serotype A strain H99 and the other for a serotype D strain JEC21, the most efficient way to study potential inter-variety chromosomal rearrangements would be to directly compare genome sequences and chromosome organizations between the two strains. Indeed, a recent comparison of the genomes of strains H99 and JEC21 identified that these two genomes were overall highly syntenic [17]. Interestingly, two large regions of high sequence identity (∼95%; likely due to introgression) and three inversions were identified between the two strains [17]. However, their criterion of sequence identity (>94%) for identifying inversions was higher than the whole-genome average. As a result, the number of inversions identified in their study is likely an underestimate. Indeed, because inversions and other types of chromosome rearrangements would likely accelerate sequence divergence between lineages relative to adjacent genomic regions, the regions around rearrangements might have lower nucleotide identity than other regions, further causing underestimates of potential rearrangements. Furthermore, the distributions of the rearrangements identified between the two sequenced genomes have not been analyzed among natural strains of *C. neoformans* to determine whether such rearrangements are strain-specific or serotype-specific. The number and distribution of rearrangements could provide valuable information for understanding the evolution of genomic architecture in *C. neoformans* and for understanding the genetic basis of partial reproductive isolation between the two varieties.

In this study, we used a set of flexible criteria to identify the number, location and distribution of chromosome rearrangements between the two varieties of *C. neoformans*. We then examined the potential polymorphisms of the non-centromeric chromosomal rearrangement regions, including both simple inversions and complex rearrangements (see below), in a collection of natural *C. neoformans* strains. We were specifically interested in the following questions. First, what types of chromosomal rearrangements are there between the two sequenced serotypes A and D genomes? And, how many unambiguous rearrangements can we detect? Second, do regions with chromosome rearrangements show lower levels of recombination frequency than those without rearrangements? And third, what is the pattern of distribution for the chromosome rearrangements among natural *C. neoformans* strains? Will we see rearrangement polymorphisms among strains within the same serotype?

## Results and Discussion

### Overall genome structure comparisons between H99 and JEC21

The two genomes that we compared, H99 (18874 kb) and JEC21 (19052 kb), were less than 1% different in size. Each of the two genomes has 14 chromosomes, and the blastn results showed that there was an overall one-to-one correspondence between chromosomes from the two genomes (Table 1). The exceptions were JEC21 chromosomes 3 and 11, which have been shown to be involved in large-scale translocations (TRs, see below). Ten of the 12 homologous chromosome-pairs had size differences that were less than 6% of the respective JEC21 chromosome. The other two chromosome-pairs showed relatively large size variations (19.6% and 22.4% for chromosome pairs involving JEC21 chromosomes 8 and 12, respectively; Table 1) due to the existence of translocations (see below).

**Table 1:** The One-to-One correspondence between chromosomes from H99 and JEC21 based on reciprocal blast searches.

JEC21		H99		% Difference^a^
Chromosome	Size (bp)	Chromosome	Size (bp)	
1	2300533	1	2291499	−0.39
2	1632307	2	1621675	−0.65
4	1783081	5	1814975	+1.79
5	1507550	6	1422463	−5.64
6	1438950	7	1399503	−2.74
7	1347793	8	1398693	+3.78
8 b	1194300	14 b	926563	−22.42 b
9	1178688	9	1186808	+0.69
10	1085720	10	1059964	−2.37
12 b	906719	4 b	1084805	+19.64 b
13	787999	12	774062	−1.77
14	762694	13	756744	−0.78
3 c	2105742	3 c	1575141	n.a. d
11 c	1019846	11 c	1561194	n.a. d
Total	19051922		18874089	−0.93

a: Percentages were calculated by dividing the size differences between the two chromosomes using the sizes of respective JEC21 chromosomes. A positive number indicates that the H99 chromosome is larger than the corresponding JEC21 chromosome. A negative number indicates that the H99 chromosome is smaller than the corresponding JEC21 chromosome.

b: Chromosomes in which there are large translocations regions.

c: Chromosomes for which homologous chromosomes are not established due to the existence of large scale of translocations.

d: Not calculated due to the existence of the large scale of translocations.

### Chromosomal rearrangements between H99 and JEC21 genomes

We found that the genome structures of JEC21 and H99 were mostly syntenic. A total of 32 chromosomal regions showed unambiguous rearrangements between these two genomes (Table 2). Lowering the sequence identity from 85% to 75% and reducing the match lengths to less than 200bp did not increase the number of unambiguous chromosome rearrangement regions (data not shown). Because the JEC21 genome has already been published while the H99 genome is not yet, we used the JEC21 chromosomes as the reference and refer to the H99 chromosomes as the rearranged types. Below we describe each of three types of chromosomal rearrangements found between the two genomes.

**Table 2:** Specific chromosomal rearrangements between H99 and JEC21 genomes.

Region^a^	JEC21 (kb)				H99 (kb)			
	Start	End	Size^b^	Adjacent to transposable element^c^	Start	End	Size^b^	Adjacent to transposable element^c^
SI(1)A	0	27	27	No	0	49	49	No
SI(1)B	852	932	80	Yes	879	960	81	Yes
SI(1)C	1874	1881	7	No	1851	1858	7	No
SI(1)D	2289	2301	12	No	2267	2291	24	Yes
SI(3)	1218	1228	10	No	771	781	10	No
SI(4)	1621	1633	12	No	1613	1623	10	Yes
SI(5)	1395	1406	11	No	1371	1381	10	No
SI(8)	846	860	14	Yes	571	588	17	No
SI(9)	721	1115	394	Yes	716	1106	390	No
CR(1)^c^	937	998	61	Yes	965	1007	42	Yes
CR(2)^c^	855	905	50	Yes	835	893	58	Yes
CR(3)^c^	745	911	166	Yes	109	235	126	Yes
CR(4)A^c^	217	279	62	Yes	233	256	23	Yes
CR(4)B	768	782	14	No	762	778	16	No
CR(4)C	1525	1621	96	Yes	1534	1613	79	Yes
CR(5)^c^	775	856	81	Yes	780	823	43	Yes
CR(6)A	75	117	42	No	69	109	40	No
CR(6)B^c^	863	939	76	Yes	828	874	46	Yes
CR(7)^c^	882	912	30	Yes	893	948	55	Yes
CR(8)^c^	706	762	56	Yes	464	485	21	Yes
CR(9)^c^	324	389	65	Yes	346	386	40	Yes
CR(10)^c^	802	879	77	Yes	829	858	29	Yes
CR(11)^c^	143	172	29	Yes	871	922	51	Yes
CR(12)^c^	129	177	48	Yes	331	376	45	Yes
CR(13)^c^	122	183	61	Yes	139	171	32	Yes
CR(14)A	45	63	18	Yes	3	26	23	Yes
CR(14)B^c^	567	645	78	Yes	579	633	54	Yes
TR(3)A	0	212	212	No	1357	1575	218	No
TR(3)B	212	1080	868	No	550	1106	556	No
TR(3)C	1815	2105	290	No	0	550	550	No
TR(8)	0	245	245	Yes	0	202	202	Yes
TR(11)	0	592	592	No	0	642	642	No

a: SI: simple inversion; CR: complex rearrangement; TR: translocation. Numbers in parentheses indicate the specific chromosomes in the JEC21 genome on which the rearrangements were located, and their corresponding chromosomes can be found in Table 1.

b: Sizes were calculated as the physical distances between the two syntenic chromosomal regions flanking the chromosomal rearrangements.

c: CR regions corresponding to the proposed centromeric regions in LOFTUS et al. [26].

### Large translocations (TR)

Karyotypic variation has been reported previously among *C. neoformans* environmental and clinical isolates [22]–[24]. Some of this variation was found among isolates from the same patient at different episodes of infection and from samples collected before and after passage in mice [21]. While the mechanisms for these observed variations were unknown, such studies suggested that translocations and large-scale deletions and duplications might occur frequently in *C. neoformans*.

**Figure 1:**
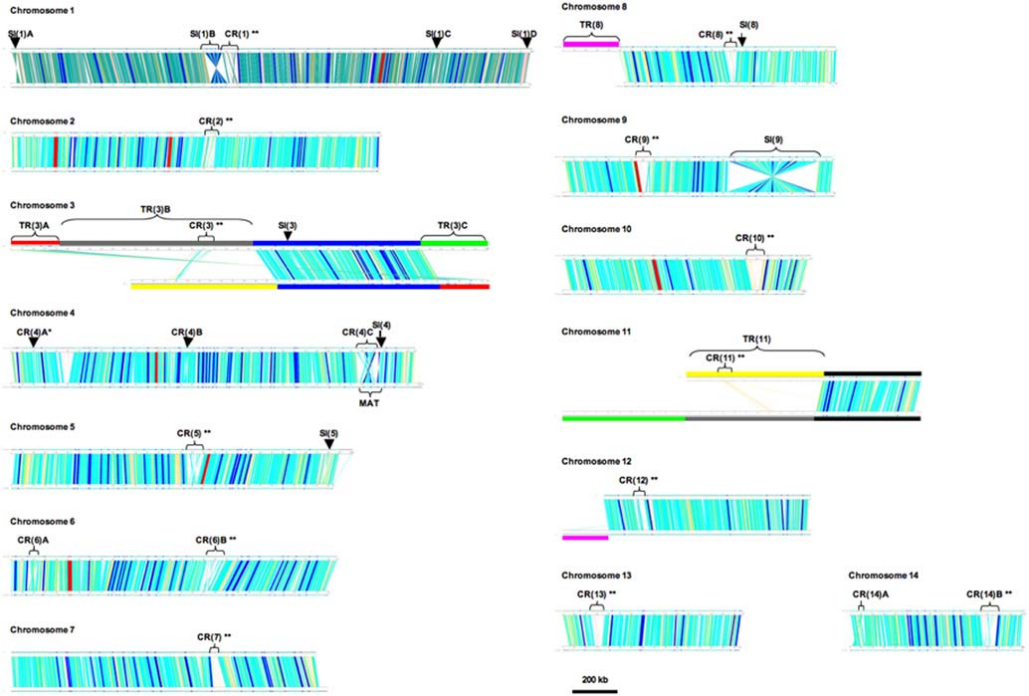
The one-to-one comparisons between chromosomes of H99 and JEC21. For each comparison, the chromosome from JEC21 is shown on top and that of H99 at the bottom. Colored lines between the paired chromosomes represent the correspondence between regions from the two chromosomes, and different colors indicate different lengths of the blast hits (red – more than 10000 bp; blue – 5000–10000 bp; light blue – 1000–5000 bp; yellow – 500–1000 bp; pink – less than 500 bp). Chromosomal rearrangements (SI, CR and TR, see MATERIALS and METHODS) are indicated in brackets. The CRs with two stars (**) are regions corresponding to proposed centromeric regions in the JEC21 genome [26]. The mating-type locus (MAT) is indicated on chromosome 4. Chromosomes 3 and 11 in the two genomes were compared, despite the existence of large-scale translocations in these chromosomes. The colored bars in chromosomes 3 and 11 correspond to those colored block arrows used in Figure 2 (see below).

Using the blast search strategy of chromosome against chromosome, we identified that five large chromosomal regions in the JEC21 genome were likely translocated in the H99 or JEC21 genomes. One translocation is intra-chromosomal, which involves a region [TR(3)A] that has been inversely translocated from the beginning to the end of the chromosome 3 (Figure 1, chromosome 3). The other four regions, located on JEC21 chromosomes 3, 8 and 11 ranging in sizes between 212kb and 868kb (Table 2 and Figure 1), did not have homologous sequences in their corresponding chromosomes, indicating that they might have been involved in inter-chromosomal translocations.

To confirm that these five TR regions were indeed translocated (i.e. they were not large insertion/deletion in one of the genomes), we used nuclear sequences of these regions from JEC21 as queries and blasted them against the whole H99 genome. The locations of the homologous regions of these “putative” chromosomal translocations in the H99 genome were determined. Our results showed that each TR region had one and only one corresponding homologous chromosomal region in the H99 genome (Figure 1, chromosomes 3, 8, 11 and 12), confirming that these TR regions were indeed translocations.

One of the four inter-chromosomal translocations occurred between chromosomes 8 and 12 [TR(8); Pink bar in Figure 1], which has been previously described by FRASER et al. [25] and has been shown to be the result of chromosomal fusion and re-breakage events that occurred during the process of producing the isogenic JEC20/JEC21 strains.

**Figure 2:**
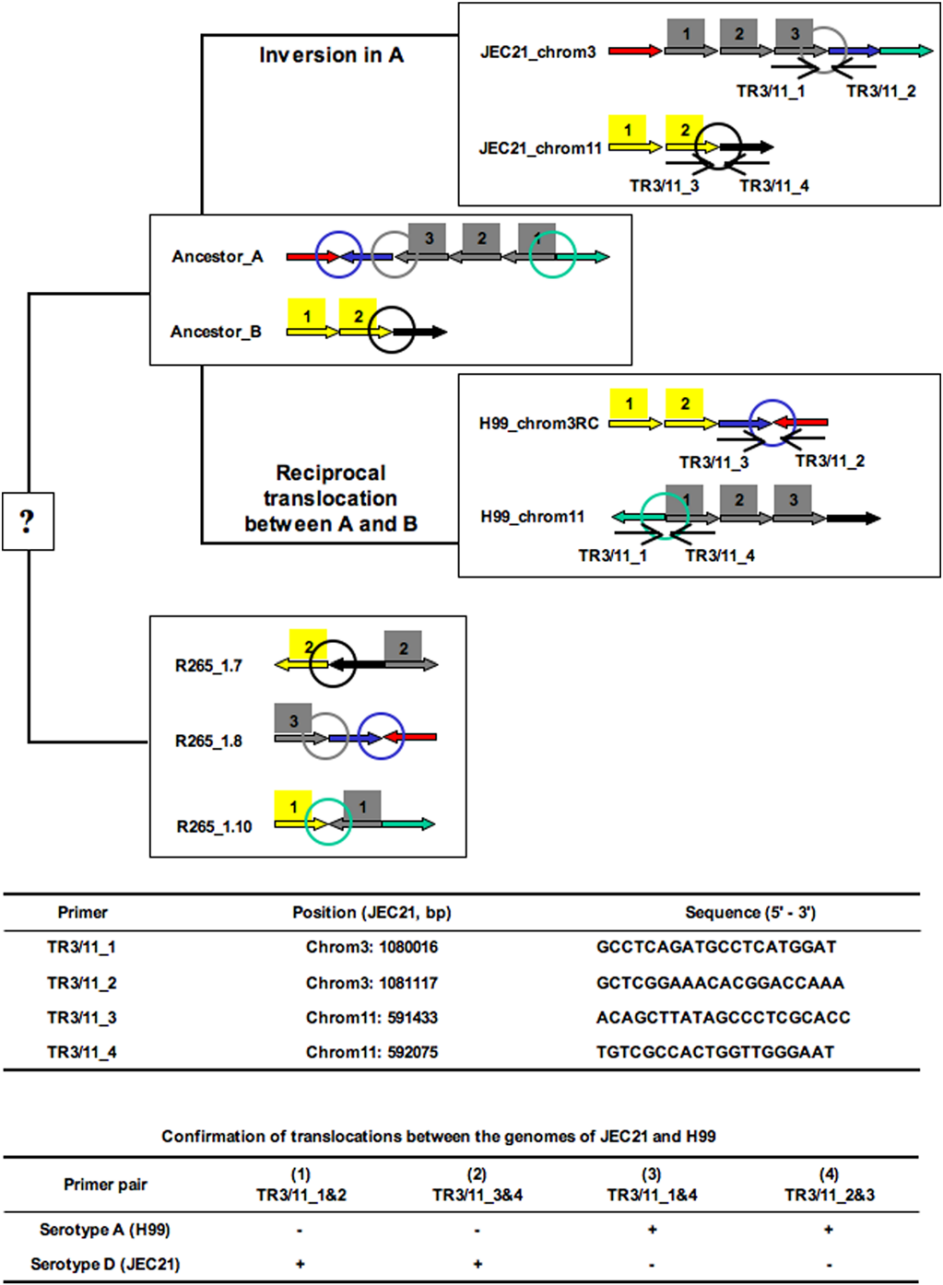
A hypothetical evolutionary history of chromosomes 3 and 11 in H99 and JEC21. The colored block arrows indicate the homologous chromosomal regions, and their relative orientations to each other, among H99, JEC21 and R265. The colors correspond to the colored bars in chromosomes 3 and 11 of Figure 1. The numbers above the block arrows represent the segments within that block. The colored circles indicate the junctions of chromosomal segments in R265 that have been found in H99 (serotype A) or JEC21 (serotype D). Arrows labeled with TR3/11_1, 2, 3 and 4 indicated the locations and orientations of the primers used for PCR confirmation of the chromosomal types of the translocation in natural isolates. The table at the bottom lists the confirmed PCR results (positive and negative) from different primer combinations in H99 and JEC21.

For two of the other three TR regions, blast hits for the middle and one end of JEC21 chromosome 3 (Figure 1, represented by grey and green bars on chromosome 3, respectively) were located at one end of chromosome 11 in the H99 genome. For the fourth translocation, one end of chromosome 11 in the JEC21 genome (Figure 1, chromosome 11 yellow bar) had significant blast hits located at one end of chromosome 3 in the H99 genome. Interestingly, the orientations of some of these chromosomal segments on the two chromosomes also differed between the two genomes. To further infer the evolutionary history of chromosomes 3 and 11, we blasted these two chromosomes in strains JEC21 and H99 against the genome sequence of strain R265 (http://www.broad.mit.edu/annotation/genome/cryptococcus_neoformans_b/Home.html), which belongs to *Cryptococcus gattii*, a closely related species of *C. neoformans*. Though the R265 genome is not completely assembled and annotated, its preliminary assembled status organized in more than 700 contigs allowed the identification of four junctions among the segments of the translocated regions. These regions are shown in Figure 2 with different colors indicating the chromosomal blocks across the three strains. The results suggest that the R265 chromosomal structures at these junctions likely represent the ancestral chromosomal structures of H99 (serotype A) and JEC21 (serotype D). Based on this inference, we propose a most parsimonious ancestral organization for chromosomes 3 and 11 and deduce the likely events that could have been responsible for generating the chromosomal structural polymorphisms between strains JEC21 and H99 (Figure 2).

Using our direct PCR strategy, we analyzed the chromosomal states at these two junctions (the black and gray circles in Figure 2) among 64 natural *C. neoformans* strains. Our results indicated all the strains had chromosomal structures identical to those of JEC21 at these two junctions. These results suggest JEC21 likely represents the ancestral states of the chromosomal structures at these two regions in *C. neoformans* and that the H99 chromosomal structures were likely generated by unique recent translocation events (Table 3).

**Table 3:** Summary of strains and their chromosome types at chromosomal rearrangement regions.

Strain	Origin	ST a	SI(1)A	SI(1)B	SI(1)C	SI(1)D	SI(3)	SI(4)	SI(5)	SI(8)	SI(9)	CR(4)B	CR(6)A	CR(14)A	TR3/11
ATCC34869	ATCC	A	A	A	A	A	A	A	A	A	A	A	A	A	D
Y195-90	BRAZIL	A	A	A	A	A^[3]^ b	A	A	A	A	A	A	A	A	D
Y288-90	CANADA	A	A^[3]^ b	A^[4]^ b	A	n.a.^c^	A	A	A	A	A	A	A	A^[11]^ b	D
Y289-90	CANADA	A	A	A	A	A	A	A	A	A	A	A	A	A	D
INN3	INDIA	A	A	A	A	A^[3]^ b	A	A	A	A	A	A	A	A	D
Y408-91	USA	A	A	A	A	A^[2]^ b	A	A	A	A	A	A	A	A	D
CDC92_001	USA	A	A	A	A	A	A	A	A	A	A	A	A	A	D
CDC92_002	USA	A	A^[3]^ b	A^[4]^ b	A	n.a.^c^	A	A^[4,5]^ b	A	A	A	A	A	A^[11]^ b	D
CDC92_003	USA	A	A	A	A	A	A	A	A	A	A	A	A	A	D
CDC92_004	USA	A	A	A	A	A	A	A	A	A	A	A	A	A	D
CDC92_007	USA	A	A^[3]^ b	A^[4]^ b	A	n.a.^c^	A	A	A	A	A	A	A	A^[11]^ b	D
CDC92_014	USA	A	A^[3]^ b	A^[4]^ b	A	n.a.^c^	A	A	A	A	A	A	A	A^[11]^ b	D
CDC92_015	USA	A	A	A	A	A^[3]^ b	A	A	A	A	A	A	A	A	D
CDC92_016	USA	A	A	A	A	A	A	A	A	A	A	A	A	A	D
CDC92_204	USA	A	A	A	A	A	A	A	A	A	A	A	A	A	D
CDC92_205	USA	A	A	A	A	A	A	A	A	A	A	A	A	A	D
CDC92_236	USA	A	A^[3,5]^ b	A	A	A	A	A	A	A	A	A	A	A	D
Y367-91	USA	A	A	A	A	A	A	A	A	A	A	A	A	A	D
Y370-91	USA	A	A	A	A	A	A	A	A	A	A	A	A	A	D
Y393-91	USA	A	A	A	A	A	A	A	A	A	A	A	A	A	D
Y490-91	USA	A	A	A	A	A	A	A	A	A	A	A	A	A	D
Y504-91	USA	A	A^[3,4]^ b	A	A	A	A	A	A	A	A	A	A	A	D
B4962	ZAIRE	A	A	A	A	A^[3]^ b	A	A	A	A	A	A	A	A	D
B4963	ZAIRE	A	A	A	A	A^[2]^ b	A	A	A	A	A	A	A	A	D
B4964	ZAIRE	A	A	A	A	A^[3]^ b	A	A	A	A	A	A	A	A	D
B4968	ZAIRE	A	A	A	A	A^[3]^ b	A	A	A	A	A	A	A	A	D
ATCC24067	ATCC	D	D	D	D	D	D	D	D	D	D	D	D^[2,3,4]^ b	D	D
ATCC34875	ATCC	D	D	D	D	D	D^[2]^ b	n.a. ^Δ^	D	D	D	D	D	D	D
Y286-90	CANADA	D	D	D	D	D	D	D	D	D	D	D	D^[2,3,4]^ b	D	D
Y290-90	CANADA	D	D	D	D	D	D	D^[1,2]^ b	D	D	D	D	D^[2,3,4]^ b	D	D
CAP67-2	USA	D	D	D	D	D	D^[2]^ b	D^[1,2]^ b	D	D	D	D	D	D	D
CDC92_027	USA	D	D	D^[1,3]^ b	D	D	D^[2]^ b	D	D	D	D	D	D	D	D
CDC92_032	USA	D	D	D	D	D	D	D	D	D	D	D	D	D	D
CDC92_076	USA	D	D	D	D	D	D	D	D	D	D	D	D^[2,3,4]^ b	D	D
CDC92_077	USA	D	D	D	D	D	D	D^[1,2]^ b	D	D	D	D	D^[2,3,4]^ b	D	D
CDC92_119	USA	D	D	D	D	D	D	D	D^[2,3]^ b	D	D	D	D^[2,3,4]^ b	D	D
CDC92_134	USA	D	D	D^[1,3]^ b	D	D	D^[2]^ b	D	D	D	D	D	D	D	D
CDC92_138	USA	D	D	D^[1,3]^ b	D	D	D^[2]^ b	D	D	D	D	D	D	D	D
CDC92_170	USA	D	D	D	D	D	D	D	D	D	D	D	D^[2,3,4]^ b	D	D
CDC92_178	USA	D	D	D	D	D^[1]^ b	D^[1]^ b	D	D	D	D	D	D	D	D
CDC92_198	USA	D	D	D	D	D	D	D^[1,2]^ b	D	D	D	D	D^[2,3,4]^ b	D	D
CDC92_337	USA	D	D	D	D	D	D	D	D	D	D	D	D^[2,3,4]^ b	D	D
Y494-91	USA	D	D	D	D	D	D	D	D	D	D	D	D	D	D
ATCC48184	ATCC	AD	AD	AD	AD	AD	AD	D^[2,3]^ b	D	AD	AD	AD	A^[7,8]^D b	A^[10,11]^ b	D
ATCC32719	ATCC	AD	D	D	D	D	AD	A	AD	D	A	AD	AD^[1,2,4]^ b	D	D
CDC92-005	USA	AD	AD	A^[4,6]^D b	AD	AD	AD	A	AD^[1,2]^ b	AD	AD	AD	AD	AD	D
CDC92-026	USA	AD	A	A	A^[4]^ b	A	AD	D^[2,3]^ b	AD	AD	AD	AD	A^[7,8]^D b	A^[9]^D^[2,5]^ b	D
CDC92-046	USA	AD	A	A	A^[4]^ b	A	AD	D^[2,3]^ b	AD	AD	AD	AD	A^[7,8]^D b	AD	D
CDC92-047	USA	AD	A	A	A^[4]^ b	A^[2,3,5]^ b	AD	D^[2,3]^ b	AD	AD	AD	AD	A^[7,8]^D b	AD	D
CDC92-062	USA	AD	A	A	A^[4]^ b	A	AD	D^[2,3]^ b	AD	AD	AD	AD	A^[7,8]^D b	AD	D
CDC92-066	USA	AD	A	A	A^[4]^ b	A^[2,3,5]^ b	AD	D^[2,3]^ b	AD	AD	AD	AD	A^[7,8]^D b	AD	D
CDC92-074	USA	AD	A	A	A^[4]^ b	A	AD	D^[2,3]^ b	D	AD	A	D	A^[7,8]^D b	AD	D
CDC92-174	USA	AD	AD	AD	A^[4]^D b	AD	AD	D^[2,3]^ b	D	AD	AD	AD	A^[7,8]^D b	AD	D
CDC92-181	USA	AD	AD	AD	AD	AD	AD	A	AD^[1,2]^ b	AD	A	AD	AD	A^[9,11]^D b	D
CDC92-190	USA	AD	AD	AD	A	A	AD	AD^[2,3]^ b	AD^[1,2]^ b	AD	AD	AD	AD^[2,3,4]^ b	AD	D
CDC92-228	USA	AD	A	A	A^[4]^ b	A	AD	D^[2,3]^ b	AD	D	AD	AD	A^[7,8]^D b	AD	D
CDC92-280	USA	AD	A	A	A^[4]^ b	A	AD	D^[2,3]^ b	AD	D	A	AD	A^[7,8]^D b	AD	D
CDC92-283	USA	AD	A	A	A^[4]^ b	A	AD	D^[2,3]^ b	AD	AD	AD	AD	A^[7,8]^D b	AD	D
CDC92-304	USA	AD	AD	AD	A^[4]^D b	AD	AD	D^[2,3]^ b	D	AD	AD	AD	A^[7,8]^D b	A^[9,11]^D b	D
CDC92-328	USA	AD	A	A	A^[4]^ b	A	AD	D^[2,3]^ b	AD	AD	AD	AD	A^[7,8]^D b	AD	D
CDC92-354	USA	AD	A	A	A^[4]^ b	A	AD	D^[2,3]^ b	AD	AD	AD	AD	A^[7,8]^D b	AD	D
CDC92-355	USA	AD	A	A	A^[4]^ b	A	AD	D^[2,3]^ b	AD	AD	AD	AD	A^[7,8]^D b	AD	D
CDC92-383	USA	AD	AD	AD	AD	A^[2,3]^D^[1]^ b	AD	n.a. c	AD^[1,2]^ b	AD	AD	D	AD	AD	D
Y520-91	USA	AD	A	A	A^[4]^ b	A	AD	D^[2,3]^ b	D	AD	AD	AD	A^[7,8]^D b	AD	D

a: ST: Serotype. Serotypes identified by traditional method.

b: Superscript number(s) within brackets refers to the numbers of primer pairs that worked for that strain at that rearrangement region (see Supplemental Figures S1, S2, S3, S4, S5, S6, S7, S8, S9, S10, S11 and S12). Character (i.e. A or D) without a superscript number indicates all the primer pairs expected to work with that strain worked.

c: No primer pair worked.

### Complex rearrangements (CR)

Our genome comparison revealed 18 chromosomal regions showing complex rearrangements between H99 and JEC21 (CRs; Table 2 and Figure 1), with each including both inversions and small translocations. The sizes of the complex rearranged regions between the two genomes varied between 13kb and 166kb (Table 2). Not surprisingly, fourteen of the 18 CRs were located in the proposed centromere regions of the chromosomes in the JEC21 genome [26], consistent with previous studies showing that centromeric regions are involved in extensive chromosomal rearrangements.

The other four CRs were located on JEC21 chromosomes 4, 6 and 14. CR(4)C was located within the mating type (MAT) locus (Figure 1) and our observation is consistent with results from previous studies showing that the MAT locus in *C. neoformans* contained extensive rearrangements [26], [27]. CR(4)B and CR(6)A each contained one inversion and one local translocation, and CR(14)A contained two inversions and two local translocations (Figures 1 and Supplemental Figures S1, S2, S3, S4, S5, S6, S7, S8, S9, S10, S11 and S12).

### Simple inversions (SI)

We identified nine chromosomal segments flanked by syntenic sequences but were in reverse orientations between the H99 and JEC21 genomes. These nine simple inversions (SIs) were all paracentric and they were located on six different chromosomes (Table 2; Figure 1). There were significant size differences among the nine SI regions. The smallest SI region, SI(1)D on chromosome 1, was only 3 kb in size and contained three genes, while the largest SI region, SI(9) on chromosome 9, was about 394 kb in size and contained 151 genes.

In the study by KAVANAUGH et al. [17], three inversions were identified between these two genomes. Two of those three inversions corresponded to two SI regions identified in our study: SI(1)A and SI(1)B. We also found one SI region in the location of the third inversion reported by KAVANAUGH et al. [17], but the size of our SI was smaller (12 kb compared to 70 kb) than they reported. The reason that we identified more SI regions was probably because the criteria used to filter blast hits in our study were more relaxed than those applied in the KAVANUGH et al. study [17]. In their study, only regions longer than 1000 bp with nucleotide identity higher than 94% were considered and inversions with lower sequence similarities and/or shorter lengths would have been missed.

The nine SIs identified between the H99 and JEC21 genomes in our study were fewer than those reported in other genome comparison studies involving other fungal species. For example, FISCHER et al. [2] found that the numbers of small inversions ranged from 59 to 773 between pairs of hemiascomycetous yeast species. They identified that the lowest number of inversions was between the most closely related species pair, *Kluyveromyces lactis* and *Ashbya gossypii*, which had 59 small inversions. One reason for the small number of inversions observed here might be because we did not include the ambiguous putative small inversions located within the four large translocations and within the CR regions [e.g. CR(4)B, Figure 1]. Another reason might be related to the length of time the compared genomes have diverged from each other. The genome pairs analyzed in the study by FISCHER et al. [2] were from different, reproductively isolated species that have probably diverged from each other for a lot longer than between varieties *grubii* and *neoformans* of *C. neoformans*.

Interestingly, though the number of inversions identified between the JEC21 and H99 genomes seemed fewer than those found between the hemiascomycetous species, the sizes of the inversions were bigger between JEC21 and H99 than those between the yeast species. Specifically, FISCHER et al. [2] found that the average numbers of genes per inversion were usually less than three in their comparisons. In contrast, the average number of genes within the 11 SI regions was ∼27 between JEC21 and H99, with one inversion, SI(9), containing 151 genes (detailed data not shown). The contrasting patterns in the number and size distributions found between this study and those in FISCHER et al. [2] suggest that inversions arising at the beginning of speciation may be relatively larger than those typically observed between the genomes that have diverged for long periods of time. Over time, other events such as hybridization and chromosomal introgression could have broken up the originally inverted regions, by double recombination, gene conversions, and/or additional inversions. Breaking large inversions into smaller ones might be favored by natural selection if these chromosome segments contained or were tightly linked to loci involved in reproductive isolation, as it has been suggested that multiple shorter inversions might have a stronger effect in suppressing recombination than one large inversion [28], [29].

### Association between chromosomal rearrangements and transposable elements

Since previous studies have suggested that transposable elements could facilitate the occurrence of chromosomal rearrangements [25], we screened the genomes of H99 and JEC21 for the existence of homologous sequences of 30 transposable elements that have been identified in *C. neoformans* so far, and some of these transposable elements had been described previously by Goodwin and Poulter ([30]; see MATERIALS AND METHODS).

**Figure 3:**
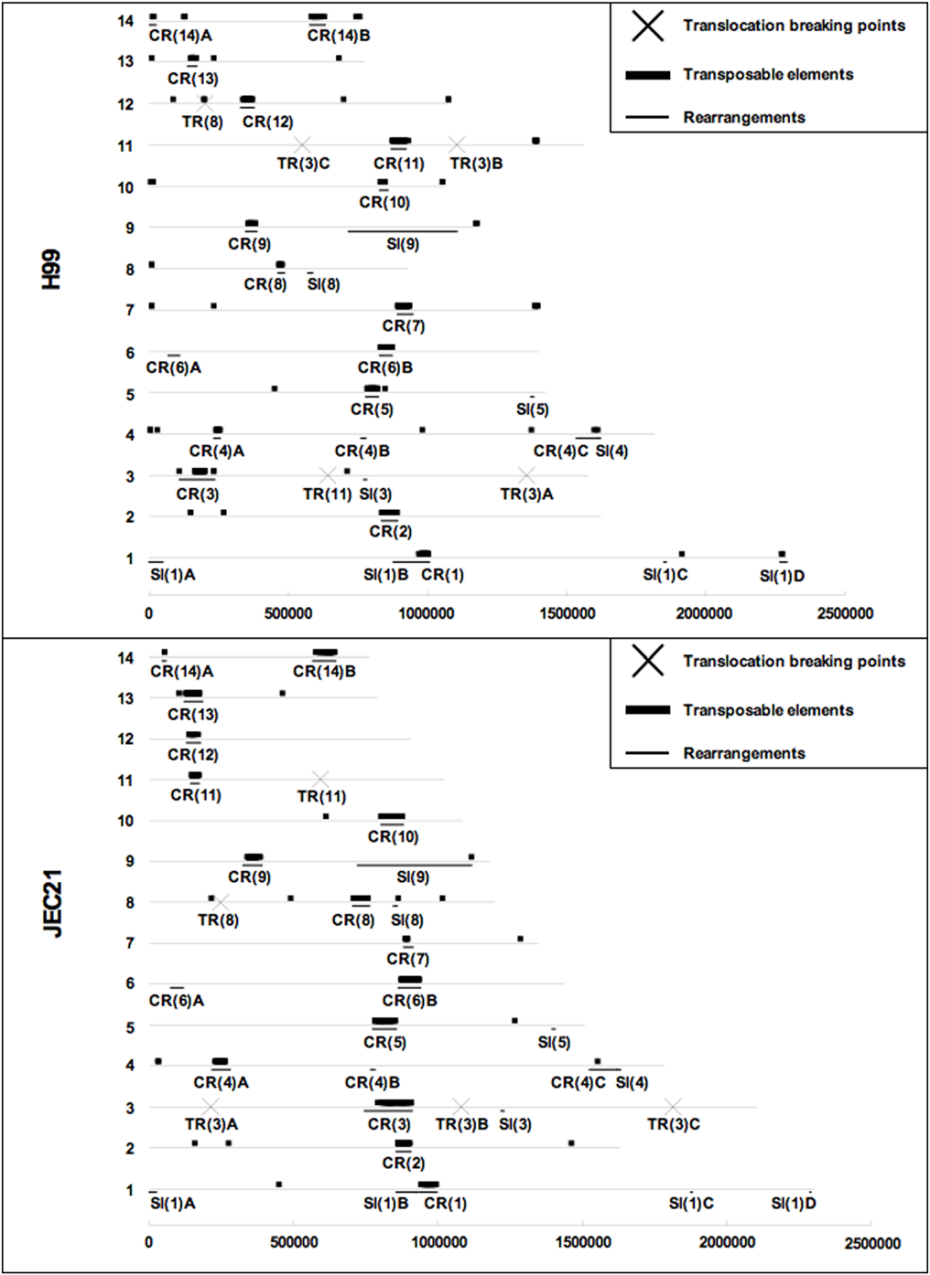
Distribution of chromosomal rearrangements reported in this study and transposable elements in the H99 and JEC21 genomes. X axis indicates the length of the chromosomes (bp). On the Y-axis, each line represents a chromosome. The black blocks above the line indicate the presence of transposable elements. The bold line segments under the line indicate the locations of chromosomal rearrangements. The crosses on the line indicate the breaking points of the translocation regions.

We found that for the boundaries of TR regions, only that of TR(8) was located in close proximity of transposable element in both H99 and JEC21 (Table 2 and Figure 3). This is consistent with the proposal in a previous study suggesting that the presence of transposable elements close to the TR(8) region might be responsible for the occurrence of that translocation event (through non-homologous recombination; [25]).

Of the 18 CR regions, all except CR(4)B and CR(6)A overlapped with the chromosomal regions that had high densities of transposable elements in both H99 and JEC21 (Table 2 and Figure 3).

While the majority of the CR regions were located close to transposable elements, only five of the nine SI regions were found to have transposable elements nearby in the H99 and/or JEC21 genome. Specifically, SI(1)B had a transposable element located in its close proximity in both the H99 and JEC21 genomes; SI(1)D and SI(4) had transposable elements located close to one of their respective boundaries in the H99 genome; and SI(8) and SI(9) had one transposable element each located close to one of their respective boundaries in the JEC21 genome (Table 2 and Figure 3).

These results suggest that simple inversions in these genomes were probably less affected by transposable elements than the CR regions. The dearth of transposable elements around SI regions with low complexity of rearrangements in comparison to the high frequency of transposable elements around CR regions with extensive rearrangement are consistent with the roles of transposable elements in facilitating chromosomal rearrangements. The potential role of transposable elements in chromosomal rearrangements is also supported by the observation that in most cases, the transposable elements identified in the close proximity of a rearrangement region belong to same type of transposons (detailed results not shown). However, it should be pointed out that factors other than transposable elements (e.g. the presence of repetitive sequences and tRNAs) could also facilitate chromosomal rearrangements.

### Low recombination frequencies surrounding the regions with CR and SI

Using the genetic linkage map constructed recently by SUN and XU [20], we calculated the recombination frequencies between each adjacent pair of markers (Table 4). In that study, the authors used 115 PCR-RFLP markers to construct a hybrid genetic linkage map between serotypes A and D *C. neoformans*. For each marker, we retrieved its physical location in the JEC21 genome [26] and genetic location in the linkage map [20]. Then for each pair of adjacent markers, the recombination frequency between them was calculated as cM/kb, i.e. the ratio of genetic distance over physical distance between the two markers. The higher the value, the more recombination events occurred per unit of physical distance, and thus the higher the recombination frequency. We excluded marker pairs for which we were not able to calculate robust cM/kb ratios. The excluded marker pairs were: i) located within the TR regions identified in the current study; or ii) located in the regions showed discrepancies between genetic linkage map and physical map in SUN and XU study ([20]; Table 4).

**Table 4:** Comparison of recombination frequencies between syntenic and rearranged regions.

Marker Pair^a^			Chromosomal Rearrangement	Physical Distance (kb)	Genetic Distance (cM)	Recombination Frequency (cM/kb*100)
CNA00050	—	CNA00670	SI(1)A	175	1.6	0.91
CNA00670	—	CNA01490		209	2.6	1.24
CNA01490	—	CNA02040		145	1.9	1.31
CNA02040	—	CNA03240		302	8	2.64
CNA03240	—	CNA04100	SI(1)B//CR(1) **	252	0.9	0.36
CNA04100	—	CNA04280		51	2.2	4.31
CNA04280	—	CNA05090		198	0.3	0.15
CNA05090	—	CNA05600		150	4.2	2.80
CNA05600	—	CNA06430		246	7.3	2.97
CNA06430	—	CNA06990	SI(1)C	153	2.9	1.89
CNA06990	—	CNA07470		145	2.9	1.99
CNA07470	—	CNA07790		107	4	3.72
CNB00700	—	CNB01310		197	1.3	0.66
CNB01310	—	CNB02080		208	1.3	0.62
CNB02080	—	CNB02980	CR(2)**	299	2.6	0.87
CNB02980	—	CNB03520		146	2.6	1.78
CNB03520	—	CNB04740		298	3.2	1.07
CNB04740	—	CNB05090		100	1.3	1.31
CNB05090	—	CNB05530		105	0	0
CNB05530	—	CNB05710		47	0.3	0.64
CND00510	—	CND01190	CR(4)A**	192	0.6	0.31
CND01190	—	CND02060		211	5	2.36
CND02060	—	CND03160	SI(4)A	299	6.1	2.04
CND03160	—	CND03480		91	1.6	1.76
CND03480	—	AD030		78	2.2	2.82
AD030	—	CND03960		74	2.2	2.96
CND03960	—	CND04540		154	2.2	1.43
CND04540	—	CND05140		157	4.3	2.75
CND05140	—	MAT	CR(4)B	165	6.2	3.76
MAT	—	CND06370	SI(4)B//CR(4)B	190	1.9	1.00
CNE00250	—	AD014		63	0.3	0.48
AD014	—	CNE01270		232	2.2	0.95
CNE01270	—	CNE01830		148	0	0
CNE01830	—	CNE02210		100	3.3	3.30
CNE02210	—	CNE03010	CR(5)**	257	2.2	0.86
CNE03010	—	CNE03700		195	1.3	0.67
CNE03700	—	AD028		128	4.3	3.35
AD028	—	CNE04300		21	0.2	0.94
CNF00290	—	CNF01350	CR(6)A	300	1.3	0.43
CNF01350	—	CNF02070		206	0.3	0.15
CNF02070	—	CNF02400		99	1.9	1.91
CNF02400	—	AD018	CR(6)B**	268	2.9	1.08
AD018	—	CNF03420		38	2.9	7.64
CNF03420	—	CNF04830		396	3.3	0.83
CNG00170	—	AD026		139	0	0
AD026	—	CNG00900		69	2.6	3.79
CNG00900	—	CNG01370		149	0	0
CNG01370	—	CNG01750		96	0	0
CNG01750	—	AD019		88	N.C. b	
AD019	—	CNG02290		67	0.3	0.45
CNG02290	—	AD020		120	N.C. b	
AD020	—	CNG03250	CR(7)**	146	0.6	0.42
CNG03250	—	CNG03900		185	2.6	1.40
CNG03900	—	AD021		64	0.6	0.93
AD021	—	CNG04610		142	0.6	0.42
CNH03700	—	CNH02750		300	N.C. c	
CNH02750	—	AD024	CR(8)**	465	4	0.86
AD024	—	CNH00030	SI(8)	371	1.6	0.43
CNI00070	—	AD006		270	5.4	2.00
AD006	—	AD005	CR(9)**	273	1.6	0.59
AD005	—	CNI02550		129	0.6	0.46
CNI02550	—	CNI02950	SI(9)	109	N.C. b	
CNI02950	—	CNI03300	SI(9)	96	0	0
CNI03300	—	CNI04370	SI(9)	269	0.6	0.22
CNJ00070	—	CNJ00540		131	2.6	1.99
CNJ00540	—	CNJ01260		209	0.6	0.29
CNJ01260	—	CNJ02080		250	2.6	1.04
CNJ02080	—	CNJ02920	CR(10)**	301	1.6	0.53-
CNJ02920	—	CNJ03090		51	0	0
CNJ03090	—	CNJ03190		44	0	0
CNL04620	—	CNL04980		108	0.3	0.28
CNL04980	—	CNL05760		206	N.C. b	
CNL05760	—	AD007		122	0	0
AD007	—	CNL06830		176	N.C. b	
AD009	—	CNM00630	CR(13)**	166	0.6	0.36
CNM00630	—	CNM01380		211	0.9	0.43
CNM01380	—	CNM01960		196	6.2	3.17
CNM01960	—	CNM02290		98	0.6	0.61
CNM02290	—	CNM02490		53	0	0
CNN00060	—	CNN00590		177	2.2	1.24
CNN00590	—	CNN01880	CR(14)A	348	N.C. c	
CNN01880	—	CNN02060	CR(14)B**	101	0.9	0.89

a: Marker pairs on JEC21 chromosomes 3 and 11 were excluded due to the existence of large scale of translocations on these chromosome.

b: Not calculated due to the inconsistence between the marker orders in the linage groups and their physical locations on the chromosome in SUN and XU [20].

c: Not calculated because the two markers are on separate linkage groups in SUN and XU [20].

We then compared these scaled recombination frequencies between those located around rearranged chromosomal regions (i.e. SI and CR regions) and those located in syntenic regions. We found that overall recombination frequencies surrounding SI and CR regions were significantly lower than the recombination frequencies in the syntenic chromosomal regions (Mann-Whitney U test, P<0.05). We did not find significant difference in recombination frequencies between marker pairs surrounding SI regions and CR regions (Mann-Whitney U test, P>0.05).

It has been suggested that the repression of recombination is achieved mainly through two processes. The first is through the function of mismatch repair systems, such as those shown in the budding yeast *S. cerevisiae* [31]–[33]. These “proof-reading” mechanisms interfere with the pairing between homologous chromosomes that have low sequence similarities during meiosis and thus suppress crossing over and recombination in these regions. The second mechanism for reduced recombination is through chromosome rearrangements, such as inversions and translocations. These rearrangements make the homologous chromosomes hard to pair with each other during meiosis. Even if crossing over occurs in these regions, recombination could result in progeny with abnormal chromosome structures (e.g. non-centromeric or di-centromeric chromosomes) and such progeny tend to be non-viable or if viable, have low fitness, thus reducing the observable recombinant genotypes and lower “effective” recombination frequency. It should be pointed out that these two processes are not mutually exclusive. Chromosomal rearrangements could facilitate sequence divergence around the rearranged regions, thus indirectly contributing to recombination repression through the mismatch repair system [5], [34]–[36]. Consistent with this hypothesis, rearranged regions have been found associated with speciation events and significantly diverged genomic sequences among many species, including humans, dogs and mice [37]–[39].

Chromosome rearrangements, especially inversions, are known to repress recombination, restrict gene flow, and play important roles in establishing new lineages/species [5]. For example, in three hybrid zones of wild sunflowers (*Helianthus*), the average frequencies of introgression across chromosomes with rearrangements were about 50% lower than those across syntenic chromosomes [9]. Similar results have been found in a study of introgression frequency among backcrossed progeny between two sunflower species, *Helianthus annuus* and *H. petiolaris*. However, in this study, the authors found that the percentages of specific genomes that had been introgressed were about 17-fold higher in collinear regions than in rearranged regions [8].

In the SUN and XU [20] study, they found that recombination occurred at a significantly lower level (overall ∼7 fold lower) in an inter-variety hybrid cross than in the intra-variety cross in *C. neoformans* [19]. The reduction in recombination frequency during serotypes A and D hybridization could be the result of combined effects of the two aforementioned processes. In this study, we found that when only syntenic chromosomal regions were considered, the average recombination frequency (unit: cM/kb*100) was 1.45, about 5 folds lower than that reported in the intra-variety cross by MARRA et al. [19]. When SI and CR regions were compared to syntenic chromosomal regions, we found that both SI and CR regions showed significantly reduced recombination frequencies in the hybrid cross in *C. neoformans*. The average recombination frequencies in the SI and CR regions were 0.98 and 0.88 respectively, which were about 32% and 39% lower than the average recombination frequency in the syntenic chromosomal regions (1.45), respectively. The differences of recombination frequencies between chromosomal rearrangements and syntenic chromosomal regions observed here were comparable to that reported in RIESEBERG et al. [9], but significantly lower than that reported in RIESEBERG et al. [8].

It should be pointed out that in our study, most of the markers used for calculation of recombination frequencies for rearrangements were not located exactly at the boundaries of the rearranged regions. In other words, the recombination frequencies calculated for the rearrangement regions included some syntenic regions, which could have led to overestimates of recombination frequencies for the rearrangement regions. Also, because we used different serotype A strains for the genome comparison (H99) and the genetic linkage map construction (CDC15), it is possible that strain CDC15 may have unique chromosomal rearrangements that differ from both H99 and JEC21, which could also lead to underestimates of recombination frequencies for the syntenic chromosomal regions. For example, in the inter-variety cross between CDC15 and JEC20, ten pairs of markers showed no recombination but appeared to be located in syntenic chromosomal regions between H99 and JEC21 (Table 4, marker pairs with zero genetic distances). It is possible that chromosomal rearrangements might be present within or around these regions in the CDC15 genome that could have suppressed recombination in these regions.

In summary, our results suggested that chromosomal rearrangements likely contributed over 30% reduction of recombination frequency in the hybrid cross between strains of serotypes A and D. In contrast, the majority of the reduction when comparing inter-variety and intra-variety crosses was likely due to other mechanisms such as the mismatch repair systems that contributed to about 5 folds reduction [19], [20].

### Distribution of chromosomal rearrangements in natural serotype A, D and AD strains

We successfully designed PCR primers from which we were able to unambiguously differentiate the two chromosomal types of H99 (serotype A) and JEC21 (serotype D) for a total of 13 rearrangement regions, including nine SIs, three CRs and one TR (Figure 2; Supplemental Figures S1, S2, S3, S4, S5, S6, S7, S8, S9, S10, S11 and S12). The 15 CR regions that were not screened were located in JEC21 either within the MAT locus [CR(4)C] or in the proposed centromeric regions [26]. All of these 15 CR regions contained extensive rearrangements and showed very low levels of sequence similarities between H99 and JEC21 (Figure 1). We were unable to find robust primers to unambiguously screen rearrangement polymorphisms among natural strains for these regions. All 13 rearranged regions that we were able to design proper primers for were screened for potential rearrangement polymorphisms in a collection of 64 natural strains of serotypes A, D and AD (Table 4).

For one translocation juncture that we screened, TR3/11, all the natural isolates had the same chromosomal type as JEC21, suggesting this translocation was an unique event that happened only in the evolution of the H99 genome (Table 4; Figure 2).

For the nine SI and three CR regions, we found that all strains belonging to serotype A had the same chromosome types and that they were different from all the serotype D strains that all had the alternate chromosome types (Table 4). Our results thus indicated that at these rearrangement regions, the two chromosomal types were likely each fixed within each of the two varieties.

Among the 21 serotype AD strains, we were able to obtain PCR products for all of the nine SI and three CR regions using at least one of the primer pairs that worked for respective serotype A or D strains. Our results indicated that the 21 serotype AD strains showed different levels of heterozygosity at different SI and CR regions (Table 4). Two rearranged regions [SI(3) and CR(6)A] showed 100% heterozygosity (i.e. all the serotype AD strains possessed both types of the chromosome). In addition, we found that no strain was homozygous for the serotype D chromosomal type in region SI(9), and that no strain was homozygous for the serotype A chromosomal type in three regions [SI(5), SI(8) and CR(4)B]. In seven of the 12 SI and CR regions, the majority of the serotype AD strains were heterozygous (more than 16 out of 21 serotype AD strains, i.e. 76%). The five exceptions were all simple inversions, with four of them being located on chromosome 1 [SI(1)A–D, all biased toward the serotype A chromosomal type (Chi-square test, P<0.05)] and the other one on chromosome 4 [SI(4), biased toward serotype D chromosomal type (Chi-square test, P<0.05)].

For several rearrangement regions, our PCR primers did not work for some of the natural strains, suggesting potential sequence divergence from the JEC21 and H99 genomes, the two genomes used as references for primer designs (Table 4). In addition, we also noted PCR fragment size polymorphisms among the natural strains for some of the rearranged junctures, suggesting the existence of small insertion(s) and/or deletion(s) for the amplified regions (Table 4, detailed data not shown).

The result that each serotype AD strain was heterozygous for chromosomal types in at least four rearrangement regions was consistent with previous studies that serotype AD strains originated from inter-variety hybridization between serotypes A and D strains [14]. The high levels of heterozygosity observed for most of the rearrangement regions analyzed here for serotype AD strains were also consistent with the low levels of recombination observed around these regions (see DISCUSSION above). However, we did not find any serotype AD strain heterozygous at all the 12 SI and CR regions. For these non-heterozygous rearrangement regions, the strain might contain either two copies of the same chromosome type or could be haploid for that specific chromosome(s) or chromosome region(s). The loss of a chromosome or chromosome segment could result from abnormal chromosomal segregation during the inter-variety hybridization or random loss during subsequent mitotic reproduction. Our data suggest that the losses of chromosomes or chromosomal segments are likely not random. Specifically, for the nine SI regions that had homozygous strains, five had chromosomal types biased toward serotype A (i.e., serotype AD strains are either heterozygous or homozygous for the serotype A chromosomal type, with the exception of strains CBS132) and the other four had chromosomal types biased toward serotype D. Another interesting observation is the very strong linkage disequilibrium among the four SI regions located on chromosome 1. All the chromosomal types for the four SI regions were significantly biased toward the serotype A type (Table 3). A propensity of serotype A chromosome 1 in serotype AD strains was recently reported by Hu et al. using a comparative genome hybridization procedure [40]. The reason for the clear biases in the chromosomal types in serotype AD strains is not known. However, the fact that the four SI regions on chromosome 1 showed strong linkage disequilibrium among serotype AD strains suggested the existence of strong epistatic interactions among loci along chromosome 1.

Copy number changes for chromosomal segments or whole chromosomes, occurring either spontaneously or as a result of hybridization, have been reported previously in many different fungi, such as *Saccharomyces cerevisiae* [41]–[43], *Candida albicans* [44]–[46] and *C. neoformans* [40]. These large-scale genetic changes may allow the organisms to adapt rapidly to new/changing environments. Indeed, many of these changes have been found to have phenotypic consequences. For example, in *C. albicans*, phenotypic variation in colony morphology, virulence, as well as drug resistance have been found to be associated with certain ploidy changes [44], [47]. Similarly, in *C. neoformans*, serotype AD strains have been shown to be more tolerant to the anti-fungal drug fluconazole [48], UV radiation [49], and high temperature [50], [51], possibly due to the diploid/aneuploid nature of their genomes.

Unlike plants and animals, serotype AD hybrids are not “evolutionary dead end,” despite producing meiotic progeny with low viability. This is because serotype AD strains can reproduce asexually and undergo somatic recombination to generate genetic variants. Such variants have the potential to influence the structure of natural *C. neoformans* populations. As was shown in previous studies, despite their recent origins [16], serotype AD strains can be prevalent in certain clinical samples [14]. It is not clear whether or not the genes located in these chromosomal rearrangements are contributing directly to these medically important traits. However, because these chromosomal rearrangements are repressing recombination during hybridization between serotypes A and D, they contribute to the production of diploid/aneuploid serotype AD strains, which may directly or indirectly influence the medically important traits.

We found that all 12 SI and CR regions had fixed chromosome types in the two varieties for strains across broad geographic areas. Some of the isolates of serotypes A and D were isolated from the same geographic area (e.g. San Francisco, detailed data not shown). These results suggest that gene flow between the two varieties was historically limited and hybridizations between them were relatively recent events, consistent with earlier observations [14], [15] and at least partially explains the significantly reduced viability of progeny produced by serotypes A and D hybridization. While there are some differences in the geographic ranges of serotypes A and D strains, they do overlap in their current geographic distributions, with both found in many regions of the world. The observed fixation of inversions between the two varieties suggests that they most likely reflect their ancient divergence, possibly due to geographic and/or ecological niche separations. Their current geographic distributions were likely due to recent dispersals by humans and other animals, resulting their hybridization and the generation of serotypes AD hybrids [14], [16].

## Materials and Methods

### *C. neoformans* genomes used for comparison

For the genome comparison, we used the whole-genome shotgun sequence information of two strains, H99 and JEC21. Strains H99 and JEC21 belonged to *C. neoformans* var. *grubii* (serotype A) and *C. neoformans* var. *neoformans* (serotype D), respectively. The genome sequence of strain H99 was downloaded from Broad Institute website at (http://www.broad.mit.edu/annotation/genome/cryptococcus_neoformans/MultiHome.html) and the nuclear genome is organized in 14 supercontigs. The genome sequence of strain JEC21 was published in 2004 [26]. The JEC21 genome also contains 14 chromosomes and is 19 Mbp in size.

### Types of chromosome rearrangements

In this study, we were mainly interested in large chromosomal rearrangements that could be identified unambiguously. We specifically looked at three types of chromosomal rearrangements.

The first type was translocation (TR). Translocations are identified when there are regions with no significant blast hits between corresponding homologous chromosomes but significant blast hits found between non-homologous chromosomes of the two genomes. The second type of chromosomal rearrangement was simple inversion (SI). As the name implies, a simple inversion is defined as one stretch of a chromosome, flanked by syntenic chromosomal regions but in reverse orientations in the H99 and JEC21 genomes. While it is relatively straightforward to infer these two types of chromosome rearrangements (TR and SI), the third type is complex and may involve multiple inversions and/or small-scale translocations. We call the third type of rearrangements complex rearrangements (CR) (see RESULTS AND DISCUSSION).

### Identification of chromosomal rearrangements between JEC21 and H99

To identify the rearrangements, we first did nucleotide blast searches (blastn) using individual chromosomes of JEC21 as separate queries and the complete H99 genome as subject database. This search identified the most likely corresponding chromosomes between the two genomes. After the one-to-one correspondence of chromosomes was established between the two genomes, we performed all-against-all blast searches (blastn), each time using one chromosome from JEC21 as a query and its corresponding chromosome from H99 as the subject database. Following JIN et al [52], our blast search results were selected using the following two criteria: (i) that the e-value of the blast hits be lower than e^−10^; and (ii) that the length of the blast hits be longer than 200bp and the sequence identity be higher than 85%. These two criteria were used to ensure stringency, identify robust blast hits, and reduce background noise. These selected blast results were then used as input files and imported into the GenomeComp program [53] to obtain graphic representations of the blast hits, following the program's instructions. In these graphic representations, colored lines connect homologous regions, and the lines connecting syntenic regions of two chromosomes are parallel. Potential chromosomal rearrangements were then identified through visual inspections. Each simple inversion (SI) would be marked by systematic crossings of the color lines connecting homologous sequences between H99 and JEC21, with all lines crossing one point of the graph. In contrast, a chromosomal region with a complex rearrangement (CR) would contain clusters of intersecting color lines in diverse orientations, with many different crossing points among the lines. A translocation (TR) was identified as a stretch of chromosomal region that did not have blast hits between the corresponding chromosomes. Reciprocal blast searches using H99 chromosomes as queries and JEC21 as the subject database were used to confirm the search results.

### Identification of transposable elements

We first retrieved sequences of 30 transposable elements from the *C. neoformans* database at TIGR website (http://www.tigr.org/tdb/e2k1/cna1/; Table 5). These 30 elements included all the transposable elements identified in *C. neoformans* so far and some have been described before [30]. Each of the 30 elements has a unique DNA sequences. These elements were clustered to nine different Tcn groups (e.g. Tcn1, Tcn2, etc) based on their overall sequence similarities (results not shown). We then used these 30 sequences as queries and compared them against the genome sequences of H99 and JEC21. The blast results were evaluated by two criteria: (i) that the e-value of the blast hits must be lower than e^−10^; and (ii) that the length of the blast hits must be longer than 30% of the query length. The locations of the transposable element sequences were then mapped onto H99 and JEC21 chromosomes (Figure 3).

**Table 5:** List of transposable elements screened in this study^a^

Locus^b^	Description/Category^c^
CNA01670	Transposable elements -Tcn760, putative
CNA03610	Transposable elements -Tcn1, putative
CNA03620	Transposable elements -Tcn3, putative
CNA03630	Transposable elements -Tcn2, putative
CNA03640	Transposable elements -Tcn4, putative
CNA03660	Transposable elements -Tcn6, putative
CNA03670	Transposable elements -Tcn3, putative
CNA03680	Transposable elements -Tcn4, putative
CNA03710	Transposable elements -Tcn7, putative
CNE02930	Transposable elements -Tcn5, putative
CNE02940	Transposable elements -Tcn1, putative
CNE02950	Transposable elements -Tcn4, putative
CNE02960	Transposable elements -Tcn6, putative
CNE02970	Transposable elements -Tcn2, putative
CNE02980	Transposable elements -Tcn6, putative
CNE02990	Transposable elements -Tcn3, putative
CNF03080	Transposable elements -Tcn2, putative
CNF03100	Transposable elements -Tcn4, putative
CNK00490	Transposable elements -Tcn6, putative
CNK00500	Transposable elements -Tcn5, putative
CNK00510	Transposable elements -Tcn3, putative
CNK00520	Transposable elements -Tcn3, putative
CNK00530	Transposable elements -LTR11, putative
CNM00500	Transposable elements -Tcn6, putative
CNM00510	Transposable elements -Tcn3, putative
CNM00520	Transposable elements -Tcn2, putative
CNM00530	Transposable elements -Tcn3, putative
CNM00550	Transposable elements -Tcn3, putative
CNM00560	Transposable elements -Tcn5, putative
CNM00570	Transposable elements -Tcn1, putative

a: The list was obtained by “Gene name search” using key words “transposable elements” at website http://www.tigr.org/tdb/e2k1/cna1/.

b: Names of loci are the same as those in the annotated JEC21 genome (Genbank AE017341-AE017353; AE017356).

c: Descriptions provided by TIGR website.

### Correlation between chromosomal rearrangements and recombination frequencies

To study whether recombination frequencies were affected by chromosomal rearrangements, we compared recombination frequencies around the rearranged regions (TR, SI and CR) to those in syntenic regions between the JEC21 and H99 genomes. We took advantage of the hybrid genetic linkage map constructed using serotype A and D hybrid progeny by SUN and XU [20] and used data presented in that study to calculate the ratio between genetic distance (cM) and physical distance (kb) for each marker pair. The simple ratio of cM/kb is used as an indicator of the amount of recombination occurring over one unit of physical distance (i.e. 1 kb). For each chromosomal rearrangement, we used the genetic distance (in cM) and the physical distance (in kb) between the two markers that were located just outside but most close to the two ends of the rearranged region to calculate the recombination frequency spanning the rearranged region (i.e. cM/kb ratio).

### Survey of rearrangement polymorphisms among natural *C. neoformans* strains

To examine whether the identified chromosomal rearrangements were strain-specific or serotype specific, we surveyed the distributions of all non-centromeric chromosomal rearrangements [26], including nine SIs, three CRs and one TR, among natural strains. To assay the 13 chromosomal rearrangements, we designed 4 to 12 PCR primers to flank the breaking points for each rearranged region. The alternative chromosomal arrangements were then determined by direct PCR using different primer combinations, with genomic DNA samples from strains H99 and JEC21 serving as positive/negative controls (depending on the primer combination). The details of the primer locations, their sequences, and the expected PCR products for different primer combinations in the 13 rearrangements are provided in the Figure 2 and Supplemental Figures S1, S2, S3, S4, S5, S6, S7, S8, S9, S10, S11 and S12. A total of 64 other natural strains of *C. neoformans* were examined. These strains were collected from five countries and consisted of 26 serotype A strains, 17 serotype D strains, and 21 serotype AD strains (Table 1). The PCR conditions, gel electrophoresis, staining and scoring followed those in our previous studies [20].

## Supporting Information

Figure S1 Illustration of the direct PCR strategy used to confirm different chromosomal types at SI(1)A. The drawing on the top shows the locations of primers used for chromosomal type determination in the two corresponding chromosomes of H99 and JEC21. The bars with uniform black or white colors indicate syntenic chromosomal regions flanking the rearranged regions. The bars with gradient colors indicated inverted chromosomal regions that have sequences in opposite directions in the two genomes. Arrows indicate the locations and directions of the primers in the two genomes. The table in the middle of each figure lists the primer sequences. The table at the bottom presents the expected and confirmed PCR results (positive and negative) of different primer combinations for H99 (serotype A) and JEC21 (serotype D).
(0.12 MB TIF)

Figure S2 Illustration of the direct PCR strategy used to confirm different chromosomal types at SI(1)B. The drawing on the top shows the locations of primers used for chromosomal type determination in the two corresponding chromosomes of H99 and JEC21. The bars with uniform black or white colors indicate syntenic chromosomal regions flanking the rearranged regions. The bars with gradient colors indicated inverted chromosomal regions that have sequences in opposite directions in the two genomes. Arrows indicate the locations and directions of the primers in the two genomes. The table in the middle of each figure lists the primer sequences. The table at the bottom presents the expected and confirmed PCR results (positive and negative) of different primer combinations for H99 (serotype A) and JEC21 (serotype D).
(0.12 MB TIF)

Figure S3 Illustration of the direct PCR strategy used to confirm different chromosomal types at SI(1)C. The drawing on the top shows the locations of primers used for chromosomal type determination in the two corresponding chromosomes of H99 and JEC21. The bars with uniform black or white colors indicate syntenic chromosomal regions flanking the rearranged regions. The bars with gradient colors indicated inverted chromosomal regions that have sequences in opposite directions in the two genomes. Arrows indicate the locations and directions of the primers in the two genomes. The table in the middle of each figure lists the primer sequences. The table at the bottom presents the expected and confirmed PCR results (positive and negative) of different primer combinations for H99 (serotype A) and JEC21 (serotype D).
(0.10 MB TIF)

Figure S4 Illustration of the direct PCR strategy used to confirm different chromosomal types at SI(1)D. The drawing on the top shows the locations of primers used for chromosomal type determination in the two corresponding chromosomes of H99 and JEC21. The bars with uniform black or white colors indicate syntenic chromosomal regions flanking the rearranged regions. The bars with gradient colors indicated inverted chromosomal regions that have sequences in opposite directions in the two genomes. Arrows indicate the locations and directions of the primers in the two genomes. The table in the middle of each figure lists the primer sequences. The table at the bottom presents the expected and confirmed PCR results (positive and negative) of different primer combinations for H99 (serotype A) and JEC21 (serotype D).
(0.12 MB TIF)

Figure S5 Illustration of the direct PCR strategy used to confirm different chromosomal types at SI(3). The drawing on the top shows the locations of primers used for chromosomal type determination in the two corresponding chromosomes of H99 and JEC21. The bars with uniform black or white colors indicate syntenic chromosomal regions flanking the rearranged regions. The bars with gradient colors indicated inverted chromosomal regions that have sequences in opposite directions in the two genomes. Arrows indicate the locations and directions of the primers in the two genomes. The table in the middle of each figure lists the primer sequences. The table at the bottom presents the expected and confirmed PCR results (positive and negative) of different primer combinations for H99 (serotype A) and JEC21 (serotype D).
(0.10 MB TIF)

Figure S6 Illustration of the direct PCR strategy used to confirm different chromosomal types at SI(4). The drawing on the top shows the locations of primers used for chromosomal type determination in the two corresponding chromosomes of H99 and JEC21. The bars with uniform black or white colors indicate syntenic chromosomal regions flanking the rearranged regions. The bars with gradient colors indicated inverted chromosomal regions that have sequences in opposite directions in the two genomes. Arrows indicate the locations and directions of the primers in the two genomes. The table in the middle of each figure lists the primer sequences. The table at the bottom presents the expected and confirmed PCR results (positive and negative) of different primer combinations for H99 (serotype A) and JEC21 (serotype D).
(0.12 MB TIF)

Figure S7 Illustration of the direct PCR strategy used to confirm different chromosomal types at SI(5). The drawing on the top shows the locations of primers used for chromosomal type determination in the two corresponding chromosomes of H99 and JEC21. The bars with uniform black or white colors indicate syntenic chromosomal regions flanking the rearranged regions. The bars with gradient colors indicated inverted chromosomal regions that have sequences in opposite directions in the two genomes. Arrows indicate the locations and directions of the primers in the two genomes. The table in the middle of each figure lists the primer sequences. The table at the bottom presents the expected and confirmed PCR results (positive and negative) of different primer combinations for H99 (serotype A) and JEC21 (serotype D).
(0.12 MB TIF)

Figure S8 Illustration of the direct PCR strategy used to confirm different chromosomal types at SI(8). The drawing on the top shows the locations of primers used for chromosomal type determination in the two corresponding chromosomes of H99 and JEC21. The bars with uniform black or white colors indicate syntenic chromosomal regions flanking the rearranged regions. The bars with gradient colors indicated inverted chromosomal regions that have sequences in opposite directions in the two genomes. Arrows indicate the locations and directions of the primers in the two genomes. The table in the middle of each figure lists the primer sequences. The table at the bottom presents the expected and confirmed PCR results (positive and negative) of different primer combinations for H99 (serotype A) and JEC21 (serotype D).
(0.11 MB TIF)

Figure S9 Illustration of the direct PCR strategy used to confirm different chromosomal types at SI(9). The drawing on the top shows the locations of primers used for chromosomal type determination in the two corresponding chromosomes of H99 and JEC21. The bars with uniform black or white colors indicate syntenic chromosomal regions flanking the rearranged regions. The bars with gradient colors indicated inverted chromosomal regions that have sequences in opposite directions in the two genomes. Arrows indicate the locations and directions of the primers in the two genomes. The table in the middle of each figure lists the primer sequences. The table at the bottom presents the expected and confirmed PCR results (positive and negative) of different primer combinations for H99 (serotype A) and JEC21 (serotype D).
(0.10 MB TIF)

Figure S10 Illustration of the direct PCR strategy used to confirm different chromosomal types at CR(4)B. The drawing on the top shows the locations of primers used for chromosomal type determination in the two corresponding chromosomes of H99 and JEC21. The bars with uniform black or white colors indicate syntenic chromosomal regions flanking the rearranged regions. The bars with uniform green colors indicate transpositions with the same sequence orientations. The bars with gradient colors indicated inverted chromosomal regions that have sequences in opposite directions in the two genomes. Arrows indicate the locations and directions of the primers in the two genomes. The table in the middle of each figure lists the primer sequences. The table at the bottom presents the expected and confirmed PCR results (positive and negative) of different primer combinations for H99 (serotype A) and JEC21 (serotype D).
(0.15 MB TIF)

Figure S11 Illustration of the direct PCR strategy used to confirm different chromosomal types at CR(6)A. The drawing on the top shows the locations of primers used for chromosomal type determination in the two corresponding chromosomes of H99 and JEC21. The bars with uniform black or white colors indicate syntenic chromosomal regions flanking the rearranged regions. The bars with uniform green colors indicate transpositions with the same sequence orientations. The bars with gradient colors indicated inverted chromosomal regions that have sequences in opposite directions in the two genomes. Arrows indicate the locations and directions of the primers in the two genomes. The table in the middle of each figure lists the primer sequences. The table at the bottom presents the expected and confirmed PCR results (positive and negative) of different primer combinations for H99 (serotype A) and JEC21 (serotype D).
(0.14 MB TIF)

Figure S12 Illustration of the direct PCR strategy used to confirm different chromosomal types at CR(14)A. The drawing on the top shows the locations of primers used for chromosomal type determination in the two corresponding chromosomes of H99 and JEC21. The bars with uniform black or white colors indicate syntenic chromosomal regions flanking the rearranged regions. The bars with uniform blue or green colors indicate transpositions with the same sequence orientations. The bars with gradient colors indicated inverted chromosomal regions that have sequences in opposite directions in the two genomes. Arrows indicate the locations and directions of the primers in the two genomes. The table in the middle of each figure lists the primer sequences. The table at the bottom presents the expected and confirmed PCR results (positive and negative) of different primer combinations for H99 (serotype A) and JEC21 (serotype D).
(0.17 MB TIF)
