# Supplementary figures and images for: Chromosomal Rearrangements between Serotype A and D Strains in Cryptococcus neoformans
Source: PLoS One. 2009 May 13;4(5):e5524. doi: 10.1371/journal.pone.0005524 (PMC2677675; doi:10.1371/journal.pone.0005524)

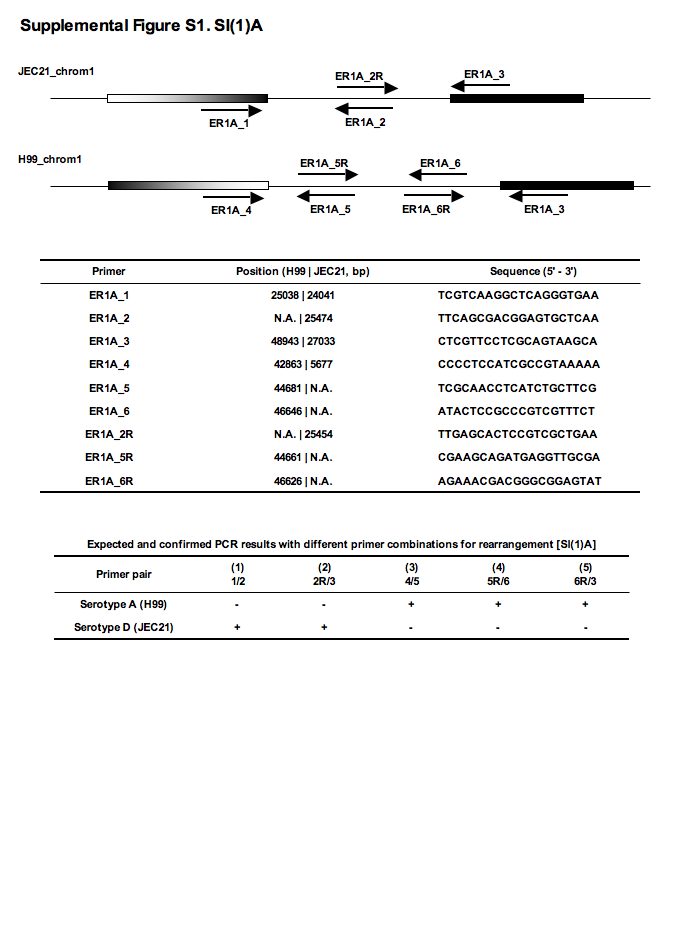

Supplement: Figure S1 — Illustration of the direct PCR strategy used to confirm different chromosomal types at SI(1)A. The drawing on the top shows the locations of primers used for chromosomal type determination in the two corresponding chromosomes of H99 and JEC21. The bars with uniform black or white colors indicate syntenic chromosomal regions flanking the rearranged regions. The bars with gradient colors indicated inverted chromosomal regions that have sequences in opposite directions in the two genomes. Arrows indicate the locations and directions of the primers in the two genomes. The table in the middle of each figure lists the primer sequences. The table at the bottom presents the expected and confirmed PCR results (positive and negative) of different primer combinations for H99 (serotype A) and JEC21 (serotype D). (0.12 MB TIF) [file pone.0005524.s001.tif]

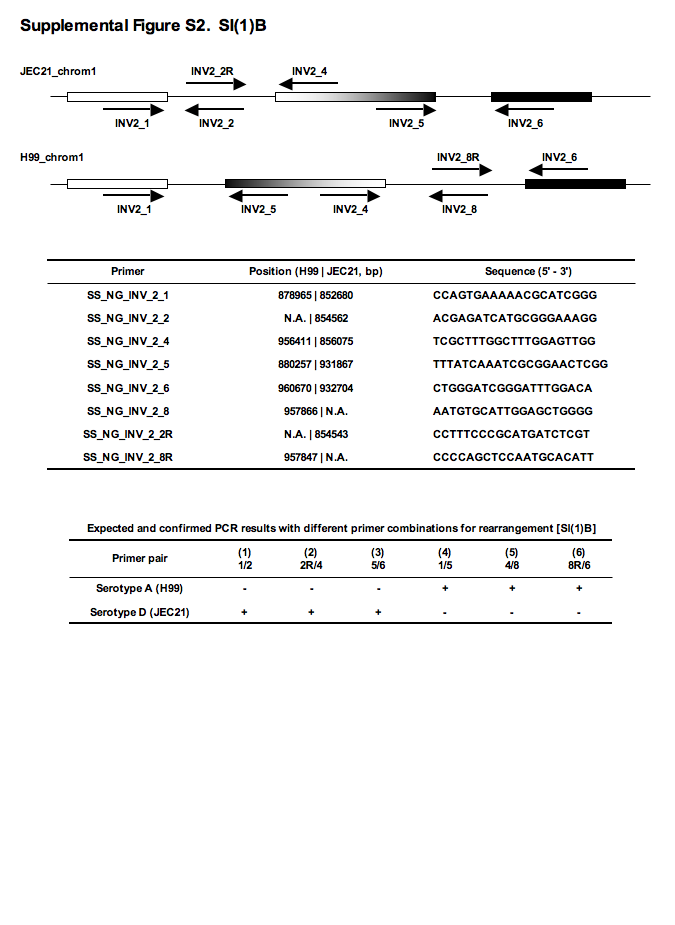

Supplement: Figure S2 — Illustration of the direct PCR strategy used to confirm different chromosomal types at SI(1)B. The drawing on the top shows the locations of primers used for chromosomal type determination in the two corresponding chromosomes of H99 and JEC21. The bars with uniform black or white colors indicate syntenic chromosomal regions flanking the rearranged regions. The bars with gradient colors indicated inverted chromosomal regions that have sequences in opposite directions in the two genomes. Arrows indicate the locations and directions of the primers in the two genomes. The table in the middle of each figure lists the primer sequences. The table at the bottom presents the expected and confirmed PCR results (positive and negative) of different primer combinations for H99 (serotype A) and JEC21 (serotype D). (0.12 MB TIF) [file pone.0005524.s002.tif]

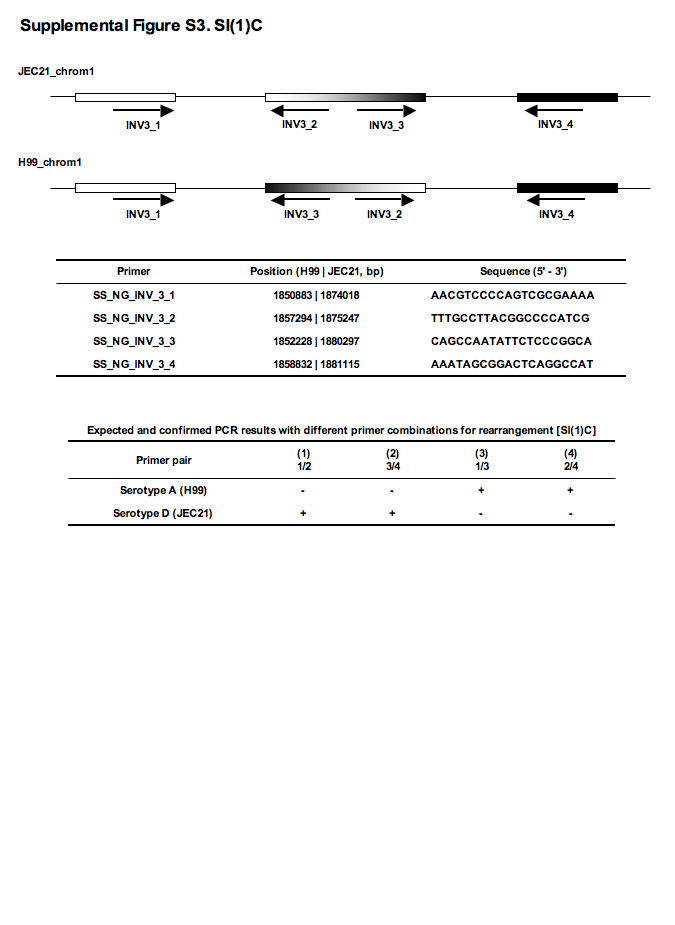

Supplement: Figure S3 — Illustration of the direct PCR strategy used to confirm different chromosomal types at SI(1)C. The drawing on the top shows the locations of primers used for chromosomal type determination in the two corresponding chromosomes of H99 and JEC21. The bars with uniform black or white colors indicate syntenic chromosomal regions flanking the rearranged regions. The bars with gradient colors indicated inverted chromosomal regions that have sequences in opposite directions in the two genomes. Arrows indicate the locations and directions of the primers in the two genomes. The table in the middle of each figure lists the primer sequences. The table at the bottom presents the expected and confirmed PCR results (positive and negative) of different primer combinations for H99 (serotype A) and JEC21 (serotype D). (0.10 MB TIF) [file pone.0005524.s003.tif]

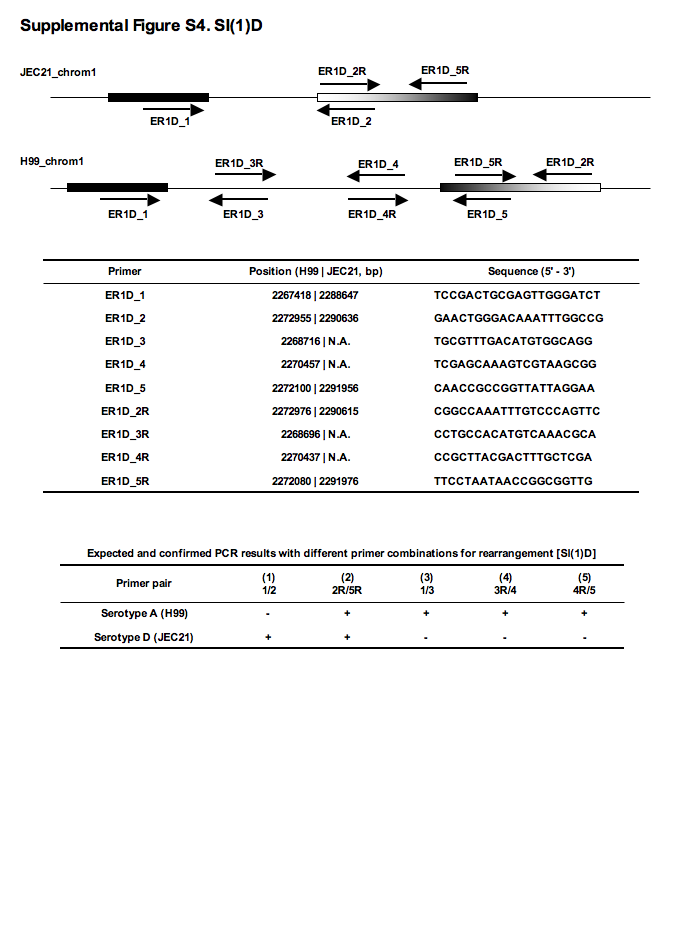

Supplement: Figure S4 — Illustration of the direct PCR strategy used to confirm different chromosomal types at SI(1)D. The drawing on the top shows the locations of primers used for chromosomal type determination in the two corresponding chromosomes of H99 and JEC21. The bars with uniform black or white colors indicate syntenic chromosomal regions flanking the rearranged regions. The bars with gradient colors indicated inverted chromosomal regions that have sequences in opposite directions in the two genomes. Arrows indicate the locations and directions of the primers in the two genomes. The table in the middle of each figure lists the primer sequences. The table at the bottom presents the expected and confirmed PCR results (positive and negative) of different primer combinations for H99 (serotype A) and JEC21 (serotype D). (0.12 MB TIF) [file pone.0005524.s004.tif]

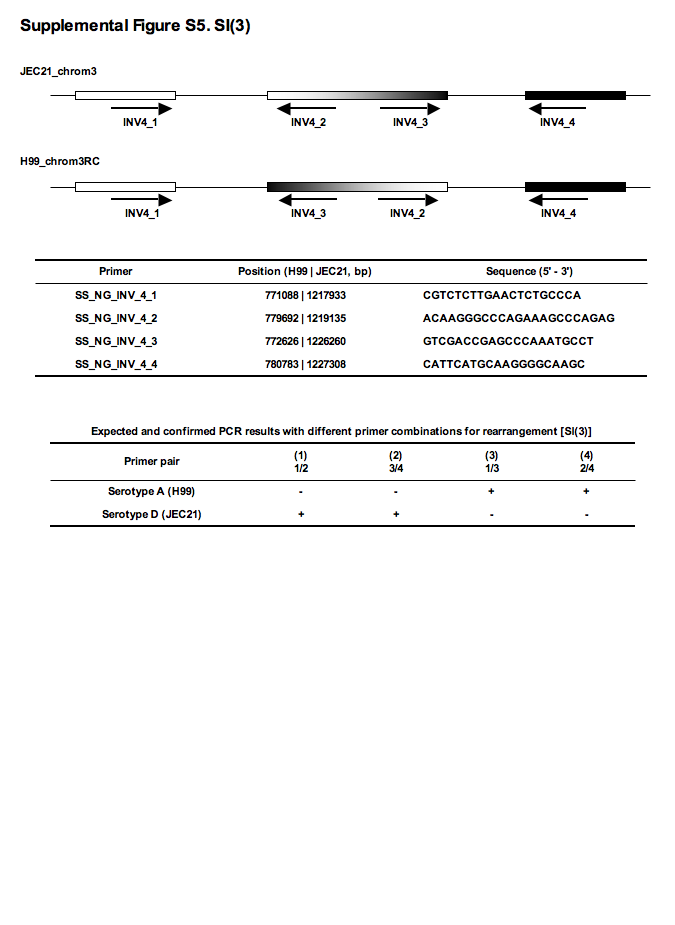

Supplement: Figure S5 — Illustration of the direct PCR strategy used to confirm different chromosomal types at SI(3). The drawing on the top shows the locations of primers used for chromosomal type determination in the two corresponding chromosomes of H99 and JEC21. The bars with uniform black or white colors indicate syntenic chromosomal regions flanking the rearranged regions. The bars with gradient colors indicated inverted chromosomal regions that have sequences in opposite directions in the two genomes. Arrows indicate the locations and directions of the primers in the two genomes. The table in the middle of each figure lists the primer sequences. The table at the bottom presents the expected and confirmed PCR results (positive and negative) of different primer combinations for H99 (serotype A) and JEC21 (serotype D). (0.10 MB TIF) [file pone.0005524.s005.tif]

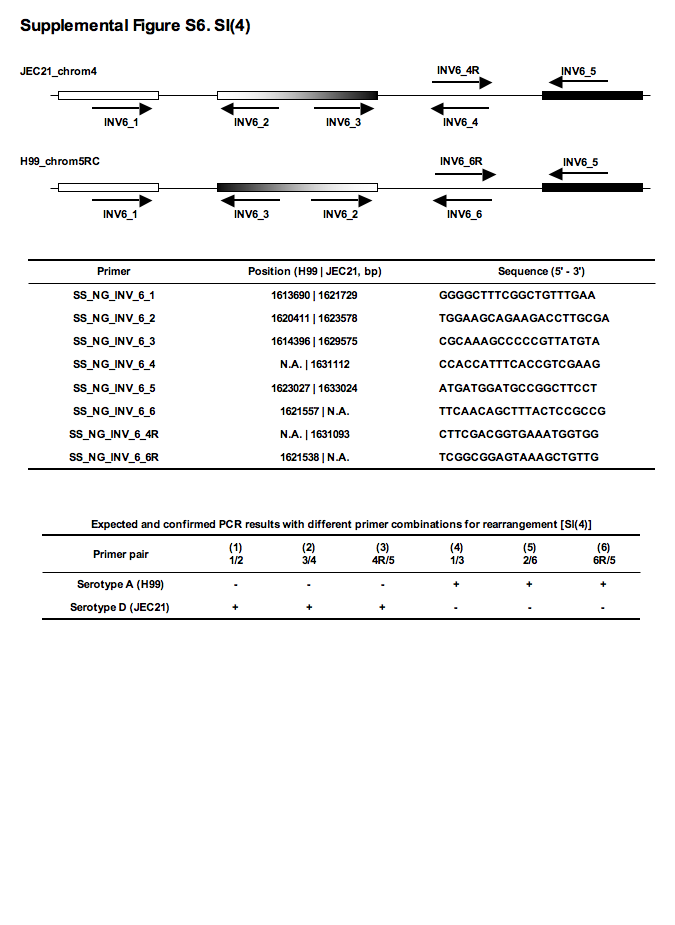

Supplement: Figure S6 — Illustration of the direct PCR strategy used to confirm different chromosomal types at SI(4). The drawing on the top shows the locations of primers used for chromosomal type determination in the two corresponding chromosomes of H99 and JEC21. The bars with uniform black or white colors indicate syntenic chromosomal regions flanking the rearranged regions. The bars with gradient colors indicated inverted chromosomal regions that have sequences in opposite directions in the two genomes. Arrows indicate the locations and directions of the primers in the two genomes. The table in the middle of each figure lists the primer sequences. The table at the bottom presents the expected and confirmed PCR results (positive and negative) of different primer combinations for H99 (serotype A) and JEC21 (serotype D). (0.12 MB TIF) [file pone.0005524.s006.tif]

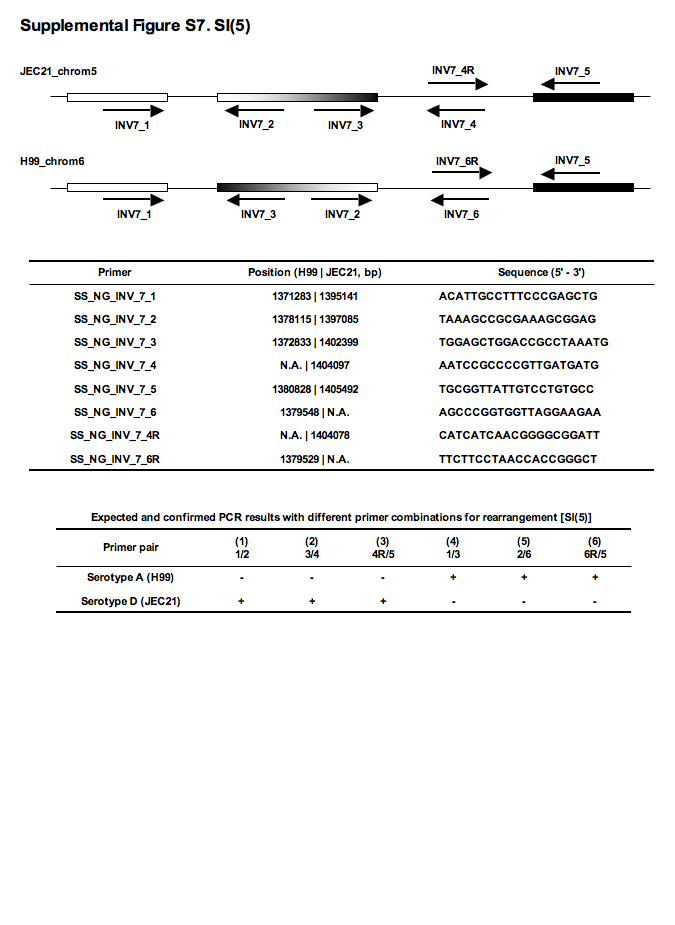

Supplement: Figure S7 — Illustration of the direct PCR strategy used to confirm different chromosomal types at SI(5). The drawing on the top shows the locations of primers used for chromosomal type determination in the two corresponding chromosomes of H99 and JEC21. The bars with uniform black or white colors indicate syntenic chromosomal regions flanking the rearranged regions. The bars with gradient colors indicated inverted chromosomal regions that have sequences in opposite directions in the two genomes. Arrows indicate the locations and directions of the primers in the two genomes. The table in the middle of each figure lists the primer sequences. The table at the bottom presents the expected and confirmed PCR results (positive and negative) of different primer combinations for H99 (serotype A) and JEC21 (serotype D). (0.12 MB TIF) [file pone.0005524.s007.tif]

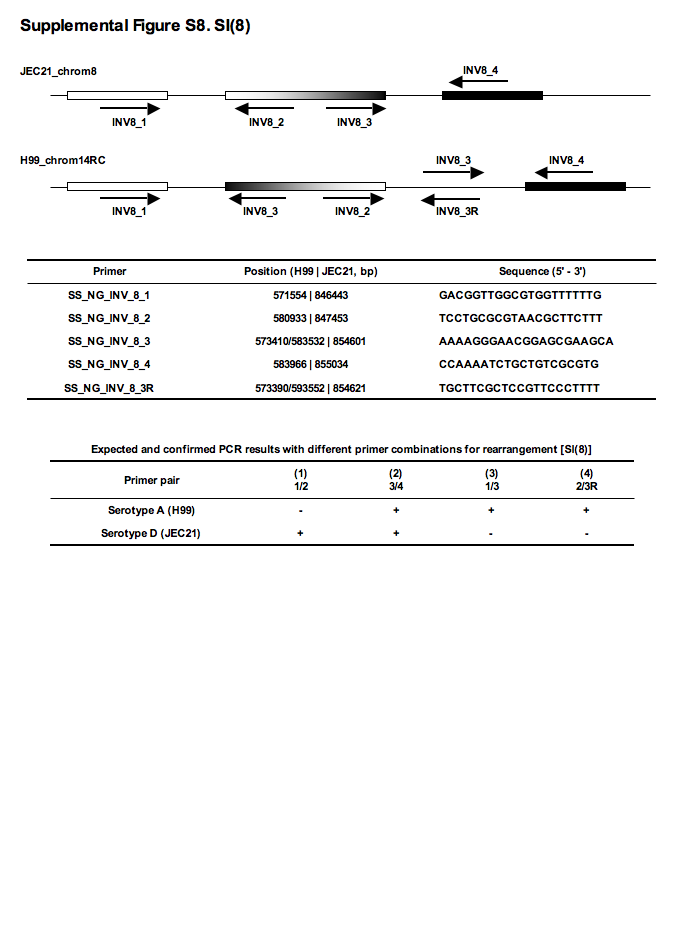

Supplement: Figure S8 — Illustration of the direct PCR strategy used to confirm different chromosomal types at SI(8). The drawing on the top shows the locations of primers used for chromosomal type determination in the two corresponding chromosomes of H99 and JEC21. The bars with uniform black or white colors indicate syntenic chromosomal regions flanking the rearranged regions. The bars with gradient colors indicated inverted chromosomal regions that have sequences in opposite directions in the two genomes. Arrows indicate the locations and directions of the primers in the two genomes. The table in the middle of each figure lists the primer sequences. The table at the bottom presents the expected and confirmed PCR results (positive and negative) of different primer combinations for H99 (serotype A) and JEC21 (serotype D). (0.11 MB TIF) [file pone.0005524.s008.tif]

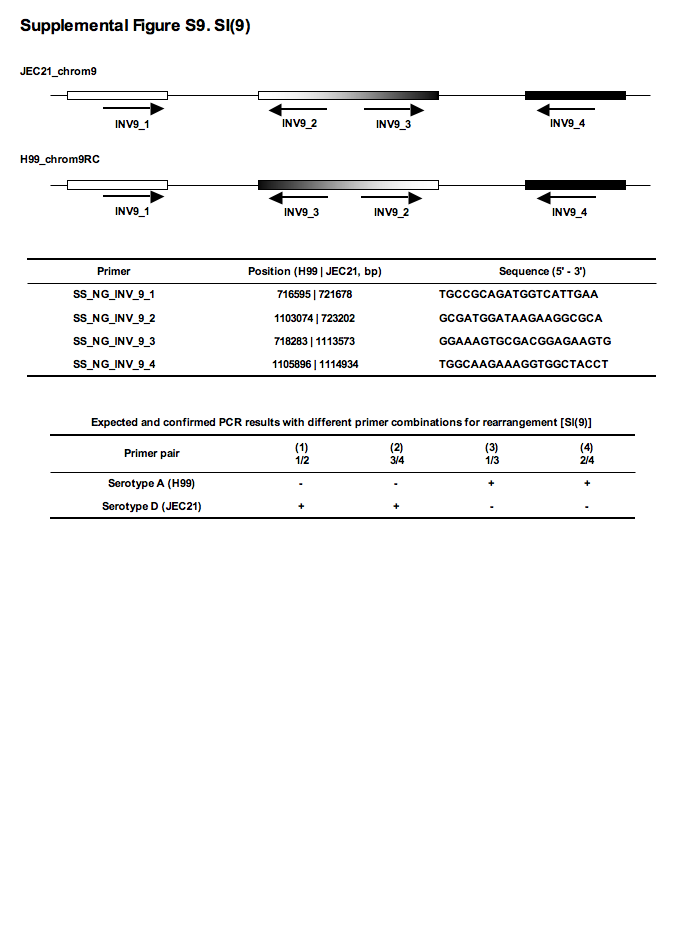

Supplement: Figure S9 — Illustration of the direct PCR strategy used to confirm different chromosomal types at SI(9). The drawing on the top shows the locations of primers used for chromosomal type determination in the two corresponding chromosomes of H99 and JEC21. The bars with uniform black or white colors indicate syntenic chromosomal regions flanking the rearranged regions. The bars with gradient colors indicated inverted chromosomal regions that have sequences in opposite directions in the two genomes. Arrows indicate the locations and directions of the primers in the two genomes. The table in the middle of each figure lists the primer sequences. The table at the bottom presents the expected and confirmed PCR results (positive and negative) of different primer combinations for H99 (serotype A) and JEC21 (serotype D). (0.10 MB TIF) [file pone.0005524.s009.tif]

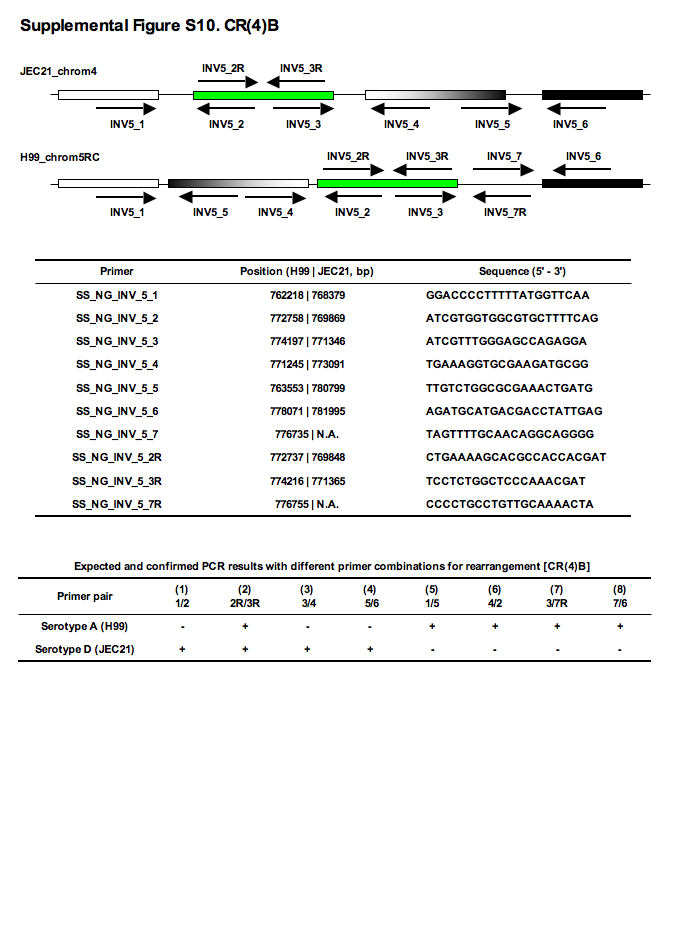

Supplement: Figure S10 — Illustration of the direct PCR strategy used to confirm different chromosomal types at CR(4)B. The drawing on the top shows the locations of primers used for chromosomal type determination in the two corresponding chromosomes of H99 and JEC21. The bars with uniform black or white colors indicate syntenic chromosomal regions flanking the rearranged regions. The bars with uniform green colors indicate transpositions with the same sequence orientations. The bars with gradient colors indicated inverted chromosomal regions that have sequences in opposite directions in the two genomes. Arrows indicate the locations and directions of the primers in the two genomes. The table in the middle of each figure lists the primer sequences. The table at the bottom presents the expected and confirmed PCR results (positive and negative) of different primer combinations for H99 (serotype A) and JEC21 (serotype D). (0.15 MB TIF) [file pone.0005524.s010.tif]

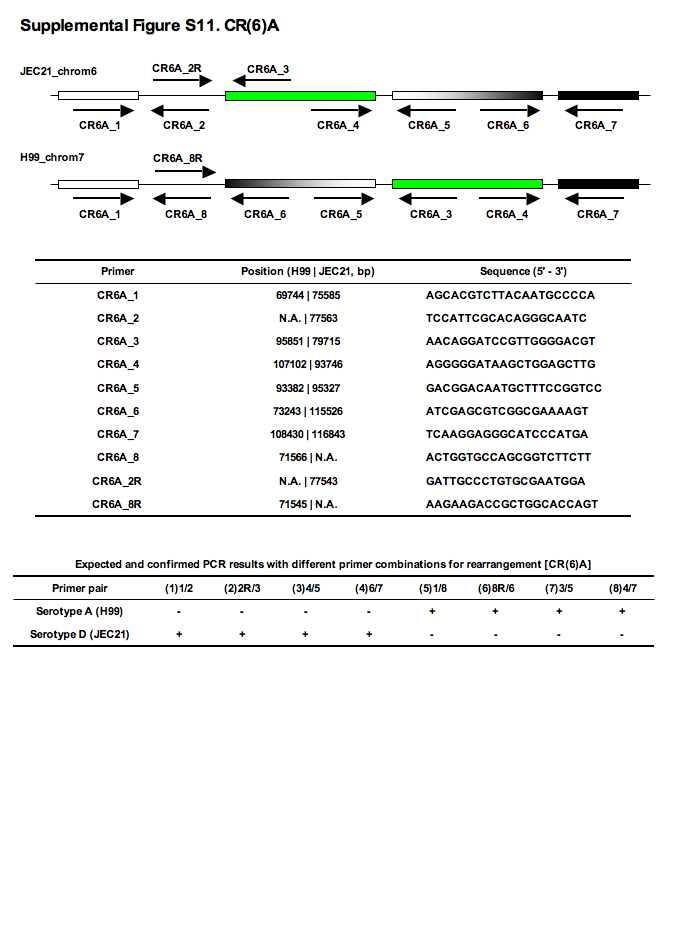

Supplement: Figure S11 — Illustration of the direct PCR strategy used to confirm different chromosomal types at CR(6)A. The drawing on the top shows the locations of primers used for chromosomal type determination in the two corresponding chromosomes of H99 and JEC21. The bars with uniform black or white colors indicate syntenic chromosomal regions flanking the rearranged regions. The bars with uniform green colors indicate transpositions with the same sequence orientations. The bars with gradient colors indicated inverted chromosomal regions that have sequences in opposite directions in the two genomes. Arrows indicate the locations and directions of the primers in the two genomes. The table in the middle of each figure lists the primer sequences. The table at the bottom presents the expected and confirmed PCR results (positive and negative) of different primer combinations for H99 (serotype A) and JEC21 (serotype D). (0.14 MB TIF) [file pone.0005524.s011.tif]

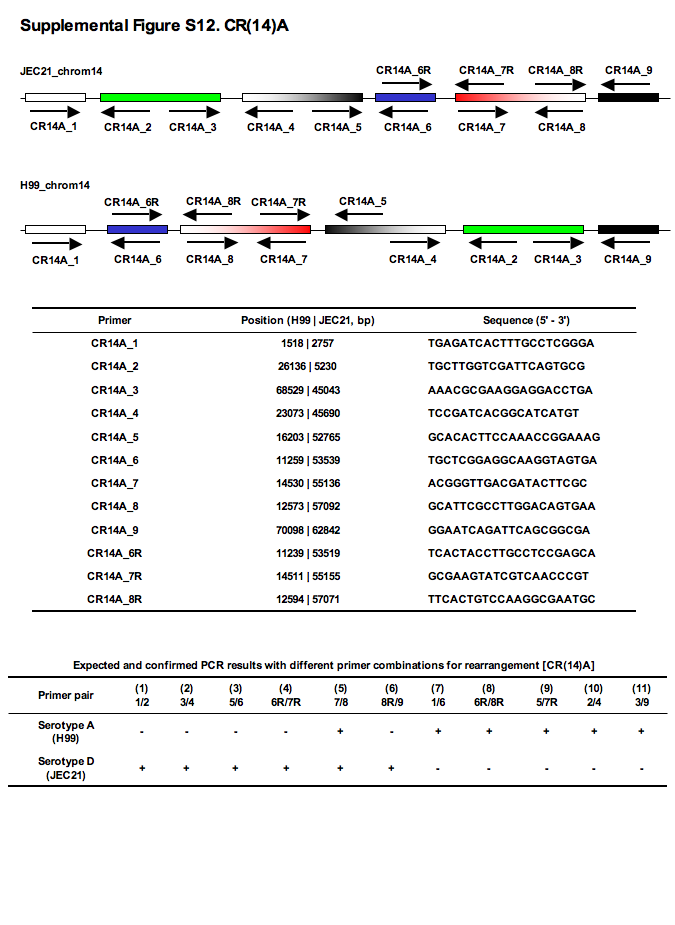

Supplement: Figure S12 — Illustration of the direct PCR strategy used to confirm different chromosomal types at CR(14)A. The drawing on the top shows the locations of primers used for chromosomal type determination in the two corresponding chromosomes of H99 and JEC21. The bars with uniform black or white colors indicate syntenic chromosomal regions flanking the rearranged regions. The bars with uniform blue or green colors indicate transpositions with the same sequence orientations. The bars with gradient colors indicated inverted chromosomal regions that have sequences in opposite directions in the two genomes. Arrows indicate the locations and directions of the primers in the two genomes. The table in the middle of each figure lists the primer sequences. The table at the bottom presents the expected and confirmed PCR results (positive and negative) of different primer combinations for H99 (serotype A) and JEC21 (serotype D). (0.17 MB TIF) [file pone.0005524.s012.tif]
